# Gliotoxin-mediated bacterial growth inhibition is caused by specific metal ion depletion

**DOI:** 10.1038/s41598-023-43300-w

**Published:** 2023-09-27

**Authors:** Shane G. Downes, Rebecca A. Owens, Kieran Walshe, David A. Fitzpatrick, Amber Dorey, Gary W. Jones, Sean Doyle

**Affiliations:** 1 Department of Biology, Maynooth University, Co. Kildare, Ireland; 2 Accuplex Diagnostics Ltd, Co. Kildare, Ireland; 3 Molecular Parasitology, University of Galway, Galway, Ireland; 4 Centre for Biomedical Science Research, School of Health, Leeds-Beckett University, Leeds, UK

**Keywords:** Antimicrobials, Applied microbiology, Bacteria, Industrial microbiology, Proteomics, Chemical biology, Microbiology

## Abstract

Overcoming antimicrobial resistance represents a formidable challenge and investigating bacterial growth inhibition by fungal metabolites may yield new strategies. Although the fungal non-ribosomal peptide gliotoxin (GT) is known to exhibit antibacterial activity, the mechanism(s) of action are unknown, although reduced gliotoxin (dithiol gliotoxin; DTG) is a zinc chelator. Furthermore, it has been demonstrated that GT synergises with vancomycin to inhibit growth of *Staphylococcus aureus*. Here we demonstrate, without precedent, that GT-mediated growth inhibition of both Gram positive and negative bacterial species is reversed by Zn^2+^ or Cu^2+^ addition. Both GT, and the known zinc chelator TPEN, mediate growth inhibition of *Enterococcus faecalis* which is reversed by zinc addition. Moreover, zinc also reverses the synergistic growth inhibition of *E. faecalis* observed in the presence of both GT and vancomycin (4 µg/ml). As well as zinc chelation, DTG also appears to chelate Cu^2+^, but not Mn^2+^ using a 4-(2-pyridylazo)resorcinol assay system and Zn^2+^ as a positive control. DTG also specifically reacts in Fe^3+^-containing Siderotec™ assays, most likely by Fe^3+^ chelation from test reagents. GSH or DTT show no activity in these assays. Confirmatory high resolution mass spectrometry, in negative ion mode, confirmed, for the first time, the presence of both Cu[DTG] and Fe[DTG]_2_ chelates. Label free quantitative proteomic analysis further revealed major intracellular proteomic remodelling within *E. faecalis* in response to GT exposure for 30–180 min. Globally, 4.2–7.2% of detectable proteins exhibited evidence of either unique presence/increased abundance or unique absence/decreased abundance (n = 994–1160 total proteins detected), which is the first demonstration that GT affects the bacterial proteome in general, and *E. faecalis*, specifically. Unique detection of components of the AdcABC and AdcA-II zinc uptake systems was observed, along with apparent ribosomal reprofiling to zinc-free paralogs in the presence of GT. Overall, we hypothesise that GT-mediated bacterial growth inhibition appears to involve intracellular zinc depletion or reduced bioavailability, and based on in vitro chelate formation, may also involve dysregulation of Cu^2+^ homeostasis.

## Introduction

Consequent to persistent overuse and abuse of antibiotics over many years, antimicrobial resistance (AMR) has developed in key bacterial pathogens. This has led to the identification of a priority group of *ESKAPE* bacterial pathogens comprising of *Enterococcus faecium*, *Staphylococcus aureus*, *Klebsiella pneumoniae*, *Acinetobacter baumannii, Pseudomonas aeruginosa* and *Enterobacter spp.*, many of which are resistant to multiple classes of antibiotics^1^. Ultimately, this has led to a reduction in the effectiveness of many established antibiotics, which has consequentially resulted in increased patient mortality. Thus, AMR has been identified as a major global threat^2^. Although next generation antibiotics, vaccination and bacteriophage therapies have been proposed to overcome AMR, the problem is still growing, which means that additional strategies must be considered to either identify new bacterial drug targets or augment existing antibiotic therapies^3–6^. Herein work is presented on the effects of the fungal non-ribosomal peptide, gliotoxin (GT), on *ESKAPE* pathogens and *Enterococcus faecalis*, selected due to the worrying trend of antibiotic resistant isolates that have been found for this bacterial species, often leading to nosocomial infections^7,8^.

Gliotoxin (GT) has a molecular mass of 326 Da, its biosynthesis is encoded by the *gli* biosynthetic gene cluster, and it is most notably produced by *Aspergillus fumigatus*^9,10^. It is a cytotoxic epipolythiodioxopiperazine (ETP) which comprises an essential intramolecular disulphide bridge, which can be converted to the dithiol form and exhibit potent Zn^2+^ chelation properties^11,12^. In addition to showing potent cytotoxicity against eukaryotic cells, GT has been shown to display broad spectrum antibiotic properties^13,14^. The accepted hypothesis has been that the oxidation and reduction of the disulphide bridge, which are key to ETP activity, produces cytotoxic reactive oxygen species^15^.

The growth inhibitory effects and mechanism of action of GT have mainly been studied in animal cells and in fungi^16–21^. It is now clear that upon entry into animal cells GT is reduced to dithiol gliotoxin (DTG) via cellular GSH, this results in ROS generation, depletion of intracellular GSH and GT efflux from cells^13^. Cell death ultimately follows. No information is available on the specific and direct impact of DTG in animal cells, although it has been demonstrated to directly inhibit the activity of LTH_4_^12^. In fungi which produce GT (e.g., *A. fumigatus*) and related ETPs, a self-protection system involving DTG oxidation to GT via oxidoreductase GliT and efflux via transporter GliA, is present^22–25^. If levels of GT/DTG increase beyond the capacity of the oxido-efflux system, then DTG is *bis*-thiomethylated to an inactive form by bis-thiomethyltransferase GtmA^26,27^. Additionally, many fungi lacking ETP biosynthetic capacity possess a *gtmA* ortholog, possibly to dissipate environmentally acquired GT or other ETPs^18,26^. DTG chelates Zn^2+^ and in *A. fumigatus*, it is now clear the GT is produced in low Zn^2+^ environments^11,28,29^. Although the precise relationship between GT/DTG and Zn^2+^ in fungi remains to be elucidated, elevated DTG levels can deplete intracellular Zn^2+^, possibly inhibit intracellular metalloenzymes and inhibit growth^11,30^. There is some evidence that fungal Zn^2+^ acquisition systems may be activated under these conditions^11,31^. Reduced holomycin, a functional dithiolopyrrolone (DTP) analogue of DTG, has been shown to inhibit bacterial metallo-β-lactamases (MBL) and proposed to alter bacterial metal homeostasis^32^.

Although there is a plethora of publications describing the inhibitory activity of GT and other ETPs against bacteria^33,34^, to the best of our knowledge, there is limited data on the GT mechanism of inhibitory action against bacterial species. Indeed, apart from a publication inferring interference with GSH levels^35^, no mechanistic details have been forthcoming about how GT inhibits bacterial growth. Interestingly, it has been demonstrated^14^ that while GT inhibited growth of a range of bacterial species and interfered with biofilm formation, culture supernatant from *A. fumigatus* Δ*gliG*, deficient in GT biosynthesis^36^, exhibited no antimicrobial activity. It has also been revealed that in addition to inhibiting growth of *Staphylococcus aureus*, GT augments the activity of vancomycin, thereby revealing synergistic activity, of unknown origin, between both antimicrobial agents^37^. Thus, although GT and other ETPs have been shown to inhibit bacterial growth, often at low µg/ml concentrations, there is a real dearth of information on how bacterial growth is inhibited.

It is not unreasonable to speculate that upon entry into bacterial cells that GT is reduced by unknown cellular reductants and that it is the reduced (DTG) form of GT which effects growth inhibition^38^. Given that Zn^2+^ chelation by DTG has been observed^11,12^, we speculate that this could also occur in bacteria, along with possible Zn^2+^ ejection from bacterial enzymes. We further hypothesise that depletion of intracellular Zn^2+^ levels would be predicted to activate uptake systems and metalloenzyme activity/stability, or abundance, could be adversely affected by GT/DTG-mediated Zn^2+^-ejection, in part as previously speculated^39^. This manuscript describes work to explore the effect of the non-ribosomal peptide GT on the Gram-positive bacterial species *E. faecalis*. Specifically, this work aims to decipher the mechanism of action behind the inhibitory activity of GT against bacteria. It is framed in the context of the reduced form of GT, dithiol gliotoxin (DTG), and its demonstrated ability to chelate zinc (Zn[DTG]).

## Results and discussion

### GT inhibits Gram positive and negative bacterial growth, which is relieved by zinc addition

**Figure 1:**
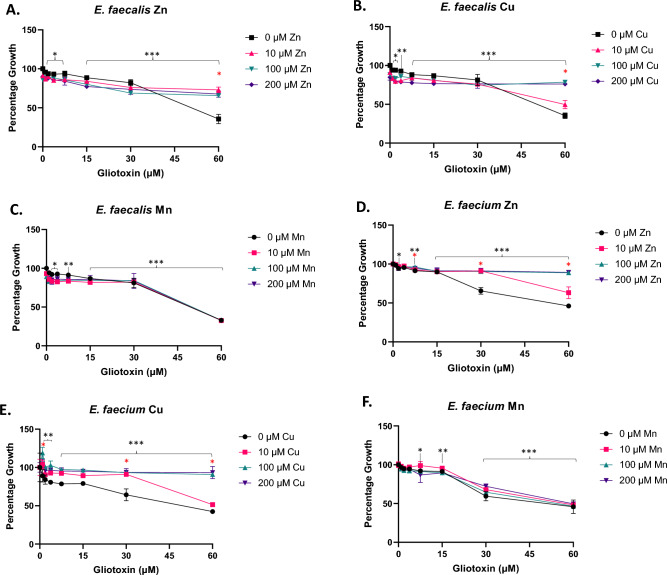
Zinc and copper relieve GT-mediated Gram positive growth inhibition. (**A**) *E. faecalis* growth in the presence of gliotoxin (0–60 µM) supplemented with Zn (0, 10, 100, or 200 µM). **(B**) *E. faecalis* growth in the presence of gliotoxin (0–60 µM) supplemented with Cu (0, 10, 100, or 200 µM). (**C**) *E. faecalis* growth in the presence of gliotoxin (0–60 µM) supplemented with Mn (0, 10, 100, or 200 µM). (**D**) *E. faecium* growth in the presence of gliotoxin (0–60 µM) supplemented with Zn (0, 10, 100, or 200 µM). (**E**) *E. faecium* growth in the presence of gliotoxin (0–60 µM) supplemented with Cu (0, 10, 100, or 200 µM). (**F**) *E. faecium* growth in the presence of gliotoxin (0–60 µM) supplemented with Mn (0, 10, 100, or 200 µM). Culture conditions: GT 15 µM = 5 µg/ml. TSB media, metals added as ZnSO_4_.7H_2_O, FeSO_4_.7H_2_O, CuCl_2_ or MnCl_2_.4H_2_O and 18 h incubation. The OD600 values equate to the following CFU/ml for *E. faecalis:* 0.2 OD600 = 1.2 × 10^8^ CFU/ml and 0.1 OD600 = 7 × 10^7^ CFU/ml. Significant inhibition due to GT exposure is indicated with black asterisks (* = *P* < 0.05, ** = *P* < 0.01, *** = *P* < 0.0001). Significant relief of inhibition at any metal concentration is shown with red asterisks (* = *P* < 0.05).

Both Gram positive species were susceptible to growth inhibition in the presence of GT. Specifically, *E. faecalis* showed significant and dose-dependent growth inhibition when grown in the presence of gliotoxin (0.9375–60 µM; *p* < 0.05–0.0001). Addition of Zn (10, 100, or 200 µM) relieved most of this inhibition at 10 µM and above Zn supplementation (*p* < 0.0001) (Fig. 1A). Cu supplementation (10, 100, or 200 µM) relieved some of this inhibition at as little as 10 µM Cu and total relief was seen at 100 and 200 µM Cu supplementation (*p* < 0.0001) (Fig. 1B). Addition of Mn (10, 100, or 200 µM) did not relieve growth inhibition (Fig. 1C). *E. faecium* showed significant and dose dependent growth inhibition when grown in the presence of gliotoxin (0.9375–60 µM) (optimally *p* < 0.0001). Zn (10, 100, or 200 µM) addition showed some relief of inhibition from 10 µM Zn and total relief was seen at 100 and 200 µM Zn supplementation (*p* < 0.0001) (Fig. 1D). Cu (10, 100, or 200 µM) also relieved growth inhibition at as little as 10 µM Cu. Total relief was seen at 100 (*p* < 0.005) and 200 µM Cu (*p* < 0.0001) supplementation (Fig. 1E), however, Mn addition (10, 100, or 200 µM) did not relieve GT-mediated growth inhibition (Fig. 1F). In *E. faecalis*, we interpret relief of GT-mediated inhibition requiring higher Cu concentration as indicative of differential biosystem dependency on this metal ion. *E. faecalis* V4932 has previously been shown to be sensitive to *A. fumigatus* GT-containing culture supernatant^33^, in accordance with our observations. As far as we can ascertain, there is only a single report of GT-mediated growth inhibition of *E. faecium*, or associated metal relief of same, in the literature^40^. Future work will be required to elucidate which Gram positive bacterial systems are subject to metallo-mediated GT inhibition.

**Figure 2:**
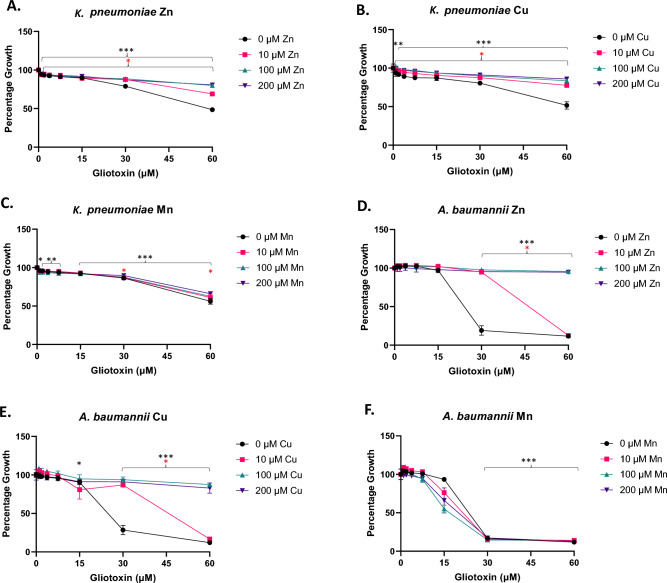
Zinc and copper relieve GT-mediated Gram negative growth inhibition. (**A**) *K. pneumoniae* growth in the presence of gliotoxin (0–60 µM) supplemented with Zn (0, 10, 100, or 200 µM). (**B**) *K. pneumoniae* growth in the presence of gliotoxin (0–60 µM) supplemented with Cu (0, 10, 100, or 200 µM). (**C**) *K. pneumoniae* growth in the presence of gliotoxin (0–60 µM) supplemented with Mn (0, 10, 100, or 200 µM). (**D**) *A. baumannii* growth in the presence of gliotoxin (0–60 µM) supplemented with Zn (0, 10, 100, or 200 µM). (**E**) *A. baumannii* growth in the presence of gliotoxin (0–60 µM) supplemented with Cu (0, 10, 100, or 200 µM). (**F**) *A. baumannii* growth in the presence of gliotoxin (0–60 µM) supplemented with Mn (0, 10, 100, or 200 µM). Culture conditions: GT 15 µM = 5 µg/ml. TSB media, metals added as ZnSO_4_.7H_2_O, FeSO_4_.7H_2_O, CuCl_2_ or MnCl_2_.4H_2_O and 18 h incubation. Significant inhibition due to GT exposure is indicated with black asterisks (* = *P* < 0.05, ** = *P* < 0.01, *** = *P* < 0.0001). Significant relief of inhibition at any metal concentration is shown with red asterisks (* = *P* < 0.05).

Both Gram negative species were also susceptible to GT-mediated growth inhibition. *K. pneumoniae* showed significant and dose-dependent growth inhibition at GT concentrations ≥ 30 µM (*p* < 0.0001). Addition of Zn (10, 100, or 200 µM) relieved most inhibition at as little as 10 µM Zn, with near total relief evident at 100 and 200 µM Zn supplementation (*p* < 0.05–0.0001) (Fig. 2A). Cu (10, 100, or 200 µM) relieved almost all this inhibition (*p* < 0.05–0.0005) (Fig. 2B), yet addition of Mn (10, 100, or 200 µM) showed no relief of inhibition (Fig. 2C). Of all bacterial species tested, growth of *A. baumannii* was most significantly inhibited in a dose-dependent manner in the presence of GT (≥ 30 µM) (*p* < 0.0001). Addition of Zn at 10 µM relieved almost all this inhibition in the presence of 30 µM GT (*p* < 0.0001) but provided no relief at 60 µM GT. Near total relief was seen at 100 and 200 µM Zn supplementation (*p* < 0.0001) (Fig. 2D). Cu at as low as 10 µM also relieved almost all growth inhibition (*p* < 0.0001), at 30 µM GT, but provided no relief at 60 µM GT. Near total relief was seen at 100 and 200 µM Cu supplementation (*p* < 0.0001)(Fig. 2E). As with all other species, Mn (10, 100, or 200 µM) did not relieve GT-mediated growth inhibition in *A. baumannii* (Fig. 2F). Experimentation showed no significant (*p* < 0.05) relief of GT-induced inhibition with supplemental Fe addition at any concentration to *E. faecalis* (Supplementary Fig. 1). This leads to the conclusion that either there is sufficient Fe present in the TSB media (5.37 µM)^41^ to overcome GT inhibition or that intracellular DTG preferentially chelates Zn or Cu. This preferential chelation may be due to the need for two DTG molecules per Fe ion to form the Fe[DTG]_2_ complex as opposed to one for Zn and Cu. ICP-MS or similar technology could be used to gain insight into the intracellular concentrations of Fe ions when the cells are challenged with GT/DTG or protected from challenge with supplementary Zn or Cu, and this will form the basis of future detailed work.

*E. faecalis* cultures grown in the presence of GT (5 µg/ml) for 1 h contained 12.6% less Zn than MeOH treated counterparts, as measured by zinquin fluorescence. To our knowledge this is the first demonstration that GT/DTG alters the internal metal abundance in bacteria, which suggests that the metal-chelating action of DTG has a negative effect on cellular growth and that GT may not be the actual active agent. This apparent Zn depletion likely plays a significant role in the inhibitory effects of GT/DTG and explains why Zn supplementation ameliorates the effects of GT. However, we observed no significant difference between intracellular Zn levels in the presence or absence of GT at 5, 15 and 30 min GT exposure which suggests that it is the bioavailability of Zn, as opposed to total intracellular Zn, which may mediate the bacterial response to GT (Supplementary Fig. 2). Importantly, the 30 min time-point overlaps with the quantitative proteomic experimentation where increased abundance of the Zn uptake receptor system is observed. This leads us to hypothesise the GT is reduced to DTG upon cellular uptake, which in turn chelates intracellular Zn thereby generating a Zn-limiting environment which activates the Zn uptake system. Future work will be focused on identifying intracellular Zn-DTG chelate and using ICP-MS to absolutely quantify intracellular Zn levels, following chelate formation and purification.

Relevantly, a range of zinc chelators, including TPEN, were evaluated for their ability to resensitize metallo-β-lactamase (MBL)-producing bacteria to antibiotics^42^. Synergistic activity between TPEN and meropenem was observed against *K. pneumoniae, Chryseobacterium indologenes, Elizabethkingia meningoseptica* and *Stenotrophomonas maltophilia* in in vitro experiments. Moreover, using the *Galleria mellonella *in vivo infection model, chelators TPEN or nitroxoline in combination with meropenem resulted in increased larval survival following infection with either *K. pneumoniae*, *E. meningoseptica* or *S. maltophilia*^42^. Thus, it is clear that artificial and naturally-occurring zinc chelators have potential to either prevent microbial growth or inactivate MBLs to overcome AMR. The demonstration of GSH-mediated activation of holomycin to reduced holomycin, with zinc-chelating ability, further underscores this biomedical application^43^.

### GT is bactericidal at high concentrations

An antibacterial agent is defined as bactericidal if it reduces the CFU/ml of a culture by more than a 3 log_10_-fold decrease. At 8 h, all GT concentrations show bactericidal activity against *E. faecalis* with a 3 log_10_-fold decrease (Supplementary Fig. 3). At 24 h, a dose-dependent recovery of the bacterial cells was observed where GT (1.875 µM) was no longer sufficient to inhibit growth. Both GT (3.75 and 7.5 µM) maintained their efficacy. GT (3.75 µM) was still effective though it fell slightly below the 3 log_10_-fold decreased cut off with 6.4 × 10^7^ CFU/ml compared to the control cultures 1.05 × 10^10^ CFU/ml. However, the highest GT concentration (7.5 µM) maintained bactericidal activity with 2.12 × 10^5^ CFU/ml.

### Zinc relieves chelator-mediated (GT and TPEN) growth inhibition of *E. faecalis* in a dose-dependent manner

**Figure 3:**
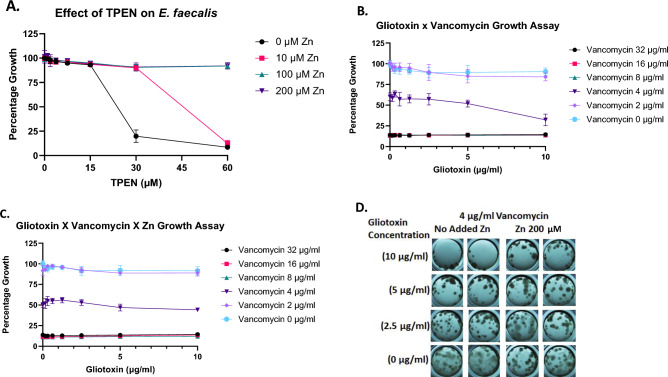
Factors influencing *E. faecali*s growth and phenotype related to Zn^2+^ availability. (**A**) Relief of TPEN-mediated growth inhibition of *E. faecalis* by Zn^2+^ addition (0—200 µM). (**B**) GT (up to 10 µg/ml) augments vancomycin-mediated growth inhibition of *E. faecalis* in a concentration-dependent manner. *E. faecalis* OD600 0.2 = 1.2 × 10^8^ CFU/ml. (**C**) Zn^2+^ (200 µM) relieves GT augmentation of vancomycin-mediated growth inhibition of *E. faecalis*. (**D**) A speckled growth phenotype of *E. faecalis* unique to the presence of vancomycin (4 µg/ml) is decreased in the presence of gliotoxin. Zn^2+^ addition (200 µM) reverses the GT dissipation effect and restores the speckled growth phenotype.

To show that metal chelation is a viable mechanism of bacterial growth inhibition, and that Zn supplementation relieves growth inhibition, the known Zn chelator TPEN was used as GT comparator. Indeed, our data shows that both molecular species chelate Zn whereby we determined the GT Zn pKd = 8.72 and TPEN Zn pKd = 9.69, respectively (Supplementary data). GT and TPEN (0–60 µM) were used to treat cultures of *E. faecalis* with and without Zn supplementation. When *E. faecalis* was grown in the presence of GT there was insignificant inhibition of growth (< 5%) at low concentrations (< 3.75 µM). The effects of GT increased with the dosage showing minor (6.1%) but significant (*p* = 0.0001) growth inhibition at 7.5 µM GT. The most significant (*p* < 0.0001) inhibition was seen at 15–60 µM GT. Inhibition rapidly increased at these concentrations 15 µM (8.0%), 30 µM (10.0%), and 60 µM (65.9%). Supplementation of 60 µM GT treated samples with all Zn concentrations showed significant (*p* < 0.0001) relief of inhibition with the percentage growth relative to the 0 µM Zn treated samples increasing by 53.3–55.8% (Supplementary Fig. 4). When *E. faecalis* was grown in the presence of TPEN significant (p = 0.0167) growth inhibition (5.1%) was seen at 7.5 µM TPEN, which became more significant (*p* = 0.0005) at 15 µM TPEN with slightly greater growth inhibition (6.9%). The most significant (*p* < 0.0001) growth inhibition was seen at 30 µM (80.1%) and 60 µM (91.5%) TPEN. Zn supplementation showed significant (*p* < 0.0001) relief of growth inhibition (70.0–71.0%). At 60 µM TPEN, 10 µM Zn was no longer able to provide significant (*p* > 0.05) relief of growth inhibition. However, 100 and 200 µM Zn supplementation provided significant (*p* < 0.0001) relief of inhibition, reducing growth inhibition by 83% (Fig. 3A). These data provide strong evidence that GT inhibits growth through intracellular Zn limitation similar to TPEN. Moreover, it is in accordance with previous work that GT/DTG, acts as a zinc chelator and either binds free zinc, or ejects it from metalloenzymes in fungi^11,29^. TPEN is a well-characterised zinc chelator, and it too appears to inhibit *E. faecalis* growth in a dose-dependent manner, which is also completely reversible by zinc addition (Fig. 3A). Combined, these data demonstrate, for the first time, a potential mechanism(s) of action, involving disruption of intracellular zinc homeostasis, for GT/DTG-mediated bacterial growth inhibition. Moreover, it complements the work of others who have deployed a range of zinc chelators (e.g., TPEN, reduced holomycin, D-alanyl-D-alanyl-D-alanine methyl ester functionalized N,N,N'-tris(2-pyridylmethyl)-ethylenediamine and tris-picolylamine (TPA) to overcome MBL degradation of target antibiotics^32,42,44,45^. Conversely, in pioneering work, microarray analysis of *E. faecalis* exposure to Zn and Cu revealed that two modules were implicated in the microbial response to metal exposure^46^. So-called module I and II, comprised genes implicated in Zn homeostasis (including the Zur transcription factor) and those involved in stress responses/basal metabolism, respectively. Relevantly, exposure to Zn resulted in downregulation of module I-encoded genes (*adcABC*/*adcA-II*) responsible for the Zn uptake system in *E. faecalis *^46^.

### GT augments vancomycin-mediated growth inhibition of ***E. faecalis***—which is relieved by Zn^2+^

Vancomycin has been shown to transiently increase expression of selected Zur regulon genes in *Streptomyces coelicolor* and also to bind to zinc in vitro^47^. Moreover, zinc has been demonstrated to induce vancomycin polymerisation thereby leading to enhanced antibiotic activity against *E. faecalis*^48^. Of further relevance is that it has been independently shown that GT acts synergistically with vancomycin, fusidic acid and linezolid, to inhibit *Staphylococcus aureus* growth^37^, an observation which suggests differential mechanistic actions and strongly underpins the rationale for further investigating that of GT/DTG. Interestingly, this previous work did not suggest a metal-dependent basis of GT-mediated inhibition^37^. However, herein we have observed that GT/DTG augments the vancomycin-mediated inhibition of *E. faecalis* growth, and that zinc addition (200 µM) negates the observed combinatorial inhibition (Fig. 3B, C). We show that GT inhibits the growth of *E. faecalis* in a dose-dependent manner, and that this inhibition is readily relieved by the addition of zinc. This implicates the disruption of microbial zinc homeostasis as a mechanism of GT antimicrobial action and provides an insight into microbial systems targeted by GT. This inhibitory effect was further explored in combination with vancomycin, the antibiotic of last resort. Checkerboard assays using GT and vancomycin showed that the presence of sub-inhibitory concentrations of GT (5 and 10 µg/ml) were sufficient to significantly (*p* < 0.03) lower the MIC of vancomycin against *E. faecalis*. This combinatorial work has also shown that GT may inhibit biofilm formation as there were apparent differences in biofilm appearance between shaking and static incubation. A unique speckled phenotype was seen at vancomycin (4 µg/ml) which was diminished with increasing GT. The presence of Zn (200 µM) attenuated the effects of GT on this phenotype (Fig. 3D). Interestingly, although GT (0.15–10 µg/ml) impeded biofilm formation in the presence of vancomycin (4 µg/ml), this inhibition did not reach statistical significance (Supplementary Fig. 5). Thus, alternative approaches will be required to further dissect any combinatorial or synergistic activity of both antimicrobials and the role of zinc in inducing vancomycin polymerisation to augment its antimicrobial activity^48^.

### DTG chelates zinc, copper and iron

**Figure 4:**
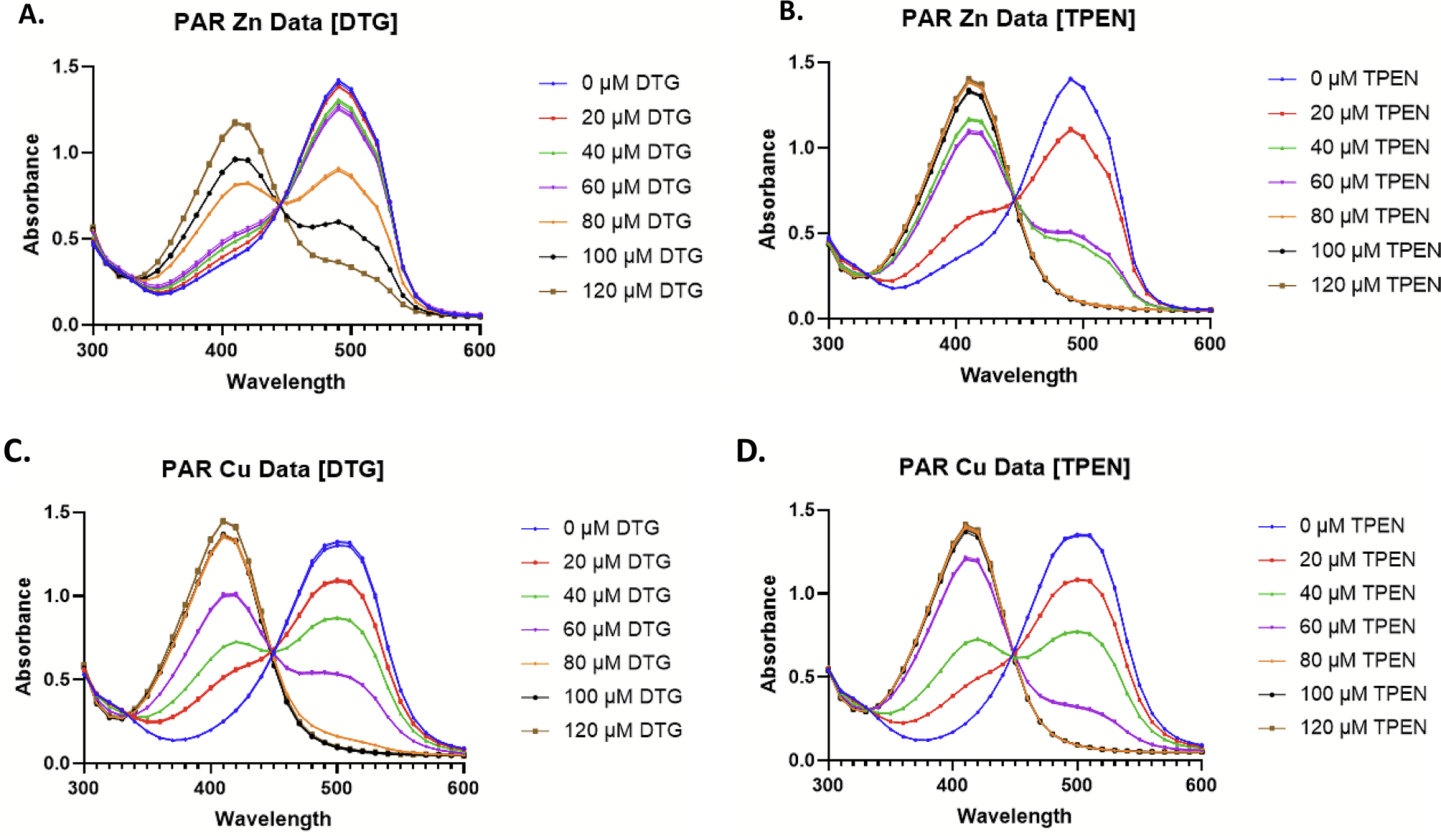
DTG ejects specific metal cations (M^2+^) from 4-(2-Pyridylazo)resorcinol (PAR). DTG removes Zn from PAR:Zn. (**A**) The effect of increasing DTG concentration on the PAR:Zn complex (495 nm). As the concentration of DTG increases the 495 nm peak decreases accompanied by an increased peak at 410 nm representing unbound PAR. (**B**) The metal chelator TPEN requires a lower concentration to remove the same amount of metal. (**C**) DTG removes Cu from PAR:Cu. The effect of increasing DTG concentration on the PAR:Cu complex (513 nm). Interestingly, DTG was able to remove Cu from PAR at lower concentrations than when removing Zn. (**D**) When this is compared to the efficacy of the metal chelator TPEN there is little difference between the two.

Using the colorimetric metal chelator 4-(2-pyridylazo)resorcinol (PAR), it was shown that DTG exhibits copper chelation properties (Fig. 4A–D). Specifically, DTG-mediated liberation of Zn (positive control;^11^) and Cu from respective PAR complexes was determined using a decrease in absorbance at 495 nm and 513 nm, respectively (Fig. 4A,C). A dose-dependent decrease in Zn(PAR)_2_ and Cu(PAR)_2_ complexes corresponding to an increase in DTG concentration was observed, indicating that DTG competitively liberates both Zn and Cu from PAR (Fig. 4A,C). Under test conditions, DTG (120 µM) dissociated most, but not all, Zn from Zn(PAR)_2_ (Fig. 4A). In contrast, TPEN removed all Zn from Zn(PAR)_2_ at concentrations ≥ 80 µM (Fig. 4B). Interestingly, DTG was more effective at dissociating Cu from Cu(PAR)_2_ with the vast majority dissociated at 80 µM and complete dissociation observed at ≥ 100 µM (Fig. 4C). DTG was only slightly less effective than TPEN at dissociating Cu from PAR, with ≥ 80 µM TPEN resulting in dissociation of all Cu from Cu(PAR)_2_ (Fig. 4D). DTG does not remove Mn from PAR chelates or Mn does not form chelates with PAR (data not shown), in accordance with previous observations regarding absence of Sporidesmin A:Mn chelates^49^. It is important to note that the formation of DTG:Zn and DTG:Cu chelates is in accordance with the observation that GT-mediated bacterial growth inhibition is reversed by these metals and not Mn, which does not appear to form a metal chelate with DTG.

**Figure 5:**
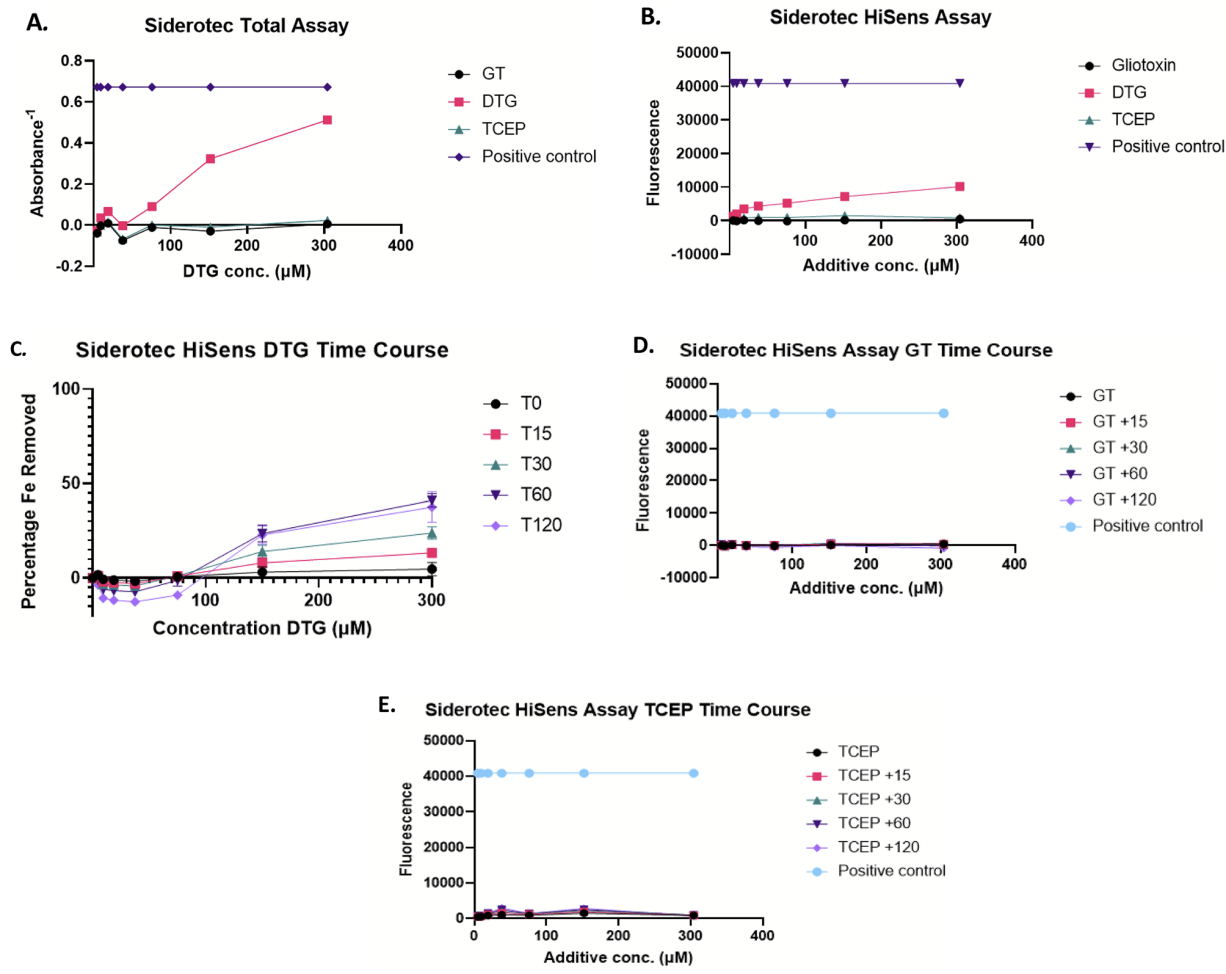
DTG specifically reacts with colorimetric and fluorescent iron chelates, possibly via Fe^3+^ reduction. DTG specifically removes Fe^3+^ from colorimetric or fluorescent Siderotec™ reagents. (**A**) The impact of gliotoxin, DTG, and TCEP on the colorimetric Fe^3+^ assay. The control (purple) indicates the signal at total Fe^3+^ removal from the chromophore. DTG (red) is the only test compound which also removes Fe^3+^. (**B**) The impact of gliotoxin, DTG, and TCEP on the fluorimetric Siderotec HiSens assay. The control (purple) indicates the signal due to Fe^3+^ removal from the fluorophore. DTG (red) is the only test compound which also removes Fe^3+^. (**C**) Temporal effect of DTG on the high sensitivity assay where individual samples were normalized to a percentage relative to the control sample (100%) at each timepoint. Peak fluorescence occurs at T60 min (41% of Fe^3+^ removed) which then begins to drop slightly by T120 min. (**D**) Neither gliotoxin or (**E**) TCEP affect the fluorometric Siderotec HiSens assay. The control (blue) indicates the signal at total Fe^3+^ removal from the fluorophore.

The colorimetric Siderotec Total assay contains a Fe^3+^ chromophore. DTG (4.7–304 µM) appeared to dissociate Fe from the chromophore in a dose-dependent manner culminating in 76% Fe removal at DTG (304 µM) after 10 min (Fig. 5A). Relevantly, neither GT or TCEP revealed any ability to dissociate Fe from the chromophore in the Siderotec Total™ assay (Fig. 5A). Furthermore, the ability of DTG to interact with Fe was also assessed using a fluorescence-based Siderotec HiSens™ assay, which confirmed a dose-dependent removal of Fe from the constituent fluorophore with increasing DTG (4.7–304 µM) (Fig. 5B). In this assay only 25% Fe was removed at 304 µM DTG, however, a temporal effect was apparent with DTG-mediated dissociation of increased amounts of Fe over time (41% Fe dissociation after 60 min, decreasing to 37.6% after 120 min at 300 µM DTG) (Fig. 5C). Again, neither GT or TCEP (4.7–300 µM) showed any notable impact on the assay at any time point, though TCEP did show slightly higher fluorescence signal than GT (Fig. 5D,E). Moreover, compared to DTG, neither GSH or DTT showed any impact on fluorescent chelate-Fe^3+^ stability in the Siderotec-HiSens™ assay (Supplementary Fig. 8), indicating the specificity of DTG for Fe^3+^ displacement from fluorescent chelates.

**Figure 6:**
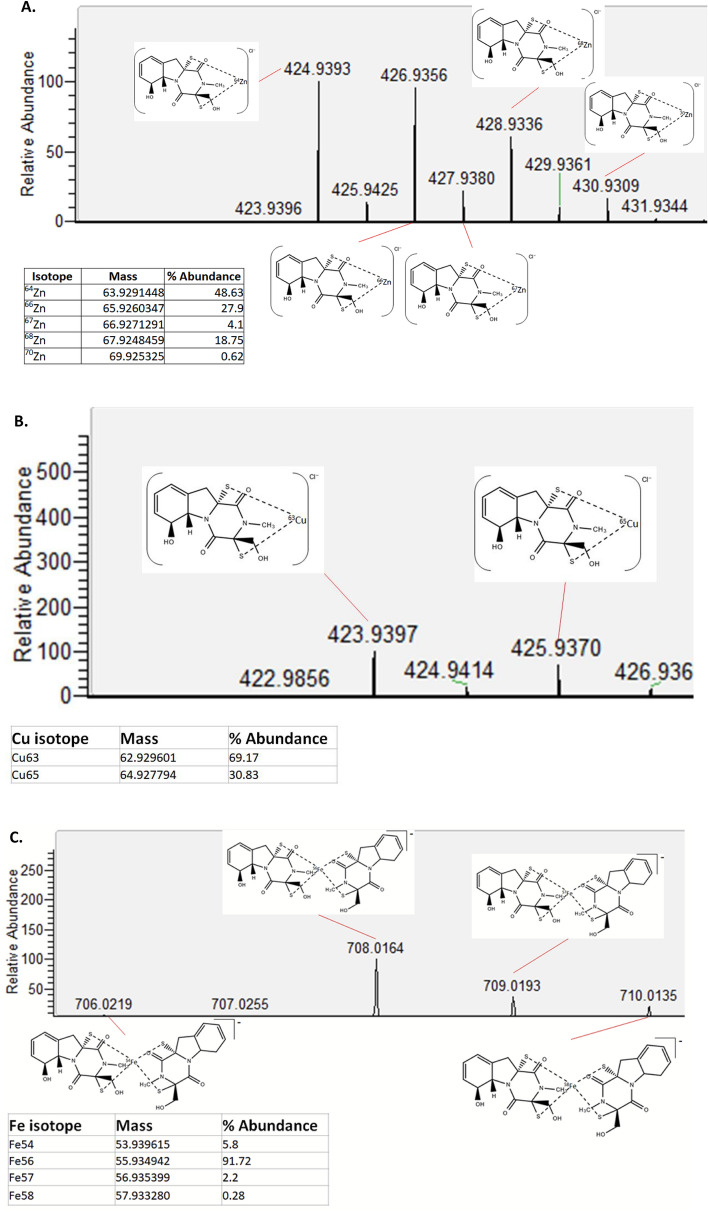
Mass spectrometric confirmation of DTG:metal ion chelates. (**A**) Identification of DTG:Zn chelate structures detected by negative mode mass spectrometry. Structures are shown with Cl^-^ ion and the relevant Zn isotope. (**B**) The DTG:Cu chelate structures detected by negative mode mass spectrometry. Structures are shown with Cl^-^ ion and the relevant Cu isotope. (**C**) The DTG:Fe chelate structures detected by negative mode mass spectrometry. Predicted structures are shown with the two DTG molecular structures and the relevant Fe isotope.

High resolution mass spectrometry in negative mode confirmed the presence of DTG:Zn (positive control), DTG:Cu, and (DTG)_2_:Fe chelates, and the resultant spectra compared to those from theoretical chelate structures (Fig. 6). The DTG:Zn chelate stoichiometry was known from previous work^11^ who found the metal adduct had an *m/z* = 424.93851, which corresponded to a 1:1 complex of DTG:Zn with a Cl^−^ ion. This structure was herein identified to exhibit *m/z* = 424.9393 ([DTG(Zn)—2H + Cl]^−^)(Fig. 6A), in accordance with previous data. The DTG:Cu chelate revealed *m/z* = 423.9397, corresponding to a 1:1 DTG:Cu chelate with Cl^-^ present ([DTG(Cu)—2H + Cl]^−^)(Fig. 6B) and the (DTG)_2_:Fe chelate showed an *m/z* = 708.0164 which corresponds to a 2:1 complex of DTG:Fe with no Cl^-^ adduct present (Fig. 6C), but with an overall single negative charge suggesting Fe^3+^ as the iron cation ([DTG_2_(Fe)—2H]^−^).

Overall, the revelation that DTG can chelate Cu and Fe is of significant biological interest, and to our knowledge has not previously been observed. It is notable that DTG:Mn chelates were not observed and suggests an element of specificity regarding the formation of DTG:Zn, DTG:Cu and (DTG)_2_:Fe chelates. Although outside the scope of the present work, the potential interaction between DTG and Fe may be of particular relevance in *A. fumigatus* and other fungi which biosynthesise ETP-type biomolecules as it suggests an additional rationale for controlling intracellular levels of DTG to avoid Fe^3+^ reduction and disrupted redox homeostasis. In *E. faecalis*, GT exposure at T = 60 min resulted in the loss of detection of ferrous transport protein A which indicates metal ion remodeling may occur due to hitherto unidentified effects of GT/DTG in bacteria. It is notable that *gliT* expression in *A. fumigatus* was reduced upon deletion of SreA, a transcriptional repressor of siderophore biosynthetic enzymes transporters to avoid excess iron uptake and resultant toxicity under iron-replete conditions^50^. This observation, in combination with our demonstration of (DTG)_2_:Fe chelate formation, suggests that GliT may potentially contribute to Fe^2+^ homeostasis in *A. fumigatus* wild-type. However, further work is undoubtedly required to fully dissect the biological significance of these observations. When a nucleophile donates lone-pair (*n*) electron density into the empty π* orbital of a nearby carbonyl group, it is referred to as an *n* → π* interaction^51^. In ETPs, the n → π* interactions can decrease the disulphide reduction potential, which may confer biologically-relevant stability on the disulphide bond in physiological environments. Indeed, it has been proposed that the two strong n → π* interactions in an ETP, like GT, can nearly completely compensate for the molecular instability caused by the strained conformation of the disulphide bond^52^. Moreover, intramolecular stability may also be affected by the hydrophobicity of the environment in which the molecule is located. Thus, we speculate that the intracellular bacterial environment is compatible with GT reduction and consequent metal ion chelation.

### GT causes significant remodelling of the *E. faecalis* proteome

Extensive alterations, including protein unique presence and increased abundance as well as unique absence and decreased abundance were observed in the *E. faecalis* proteome following GT addition (5 µg/ml) for 30, 60 and 180 min respectively (Tables 1, 2, 3, 4, 5, 6, 7, 8, 9, 10, 11, 12). Overall, between T = 30–180 min GT exposure, 4.2–7.2% of detectable proteins exhibited evidence of either unique presence/increased abundance or unique absence/decreased abundance, where total proteins detected ranged from 994 to 1160 across all analyses. To our knowledge, this is the first demonstration that GT affects the bacterial proteome in general, and *E. faecalis*, specifically.

**Table 1:** Proteins which are uniquely present in *E. faecalis* when grown in the presence of gliotoxin (5 µg/ml), compared to identical cultures grown without gliotoxin (5 µg/ml). Grown using tryptic soy broth (TSB) media for 30 min in the log phase (0.3–0.4 OD600).

Protein description	Fold Change (log2)	*p*-value	Peptides	Sequence coverage [%]	Protein IDs
Pyridine nucleotide-disulphide oxidoreductase	Unique	N/A	12	36.1	H7C719
50S ribosomal protein L33 4	Unique	N/A	2	40.8	P59629
Cobalamin synthesis protein/P47K family protein	Unique	N/A	6	25.6	Q82Z69
30S ribosomal protein S14 1	Unique	N/A	1	13.5	Q8KU58
Protein EbsA	Unique	N/A	3	19	P36920
3-dehydroquinate dehydratase	Unique	N/A	3	17.4	P36923
Protein of unknown function (DUF3114)	Unique	N/A	2	7.3	Q82YY0
Uncharacterized protein	Unique	N/A	2	18.3	Q82ZK3
Transcriptional antiterminator, bglG family	Unique	N/A	3	8.8	Q82ZT1
Xanthine/uracil permease family protein	Unique	N/A	2	8	Q82ZW2
tRNA(Met) cytidine acetate ligase	Unique	N/A	3	13.3	Q830C2
PTS system, beta-glucoside-specific IIABC component	Unique	N/A	3	6.9	Q831B4
Uncharacterized protein	Unique	N/A	2	55.9	Q834Y7
Histidine kinase	Unique	N/A	3	9.7	Q837B6
Abhydrolase_3 domain-containing protein	Unique	N/A	3	14.3	Q838Q5
Glyoxylase family protein*	Unique	N/A	3	11.2	Q834I3

*This protein exhibited overall increased abundance > 1.5-fold and was only detectable in 1 of 3 untreated samples.

**Table 2:** Proteins which are increased in abundance in *E. faecalis* when grown in the presence of gliotoxin (5 µg/ml), compared to identical cultures grown without gliotoxin (5 µg/ml). Grown using tryptic soy broth (TSB) media for 30 min in the log phase (0.3–0.4 OD600).

Protein description	Fold Change (log2)	*p*-value	Peptides	Sequence coverage [%]	Protein IDs
Adhesion lipoprotein (adcA-II)	7.28739	0.000462756	15	39.3	Q82Z67
ABC transporter, ATP-binding protein	4.10372	0.00277729	6	35.7	Q839U4
Adhesion lipoprotein (adcABC)	2.41358	0.00528037	9	45.1	Q839U5
Transcriptional regulator, Fur family	1.6883	0.00314565	4	27.8	Q831T2
Acyl carrier protein	1.27213	0.00210808	2	44.3	Q82ZE9
Copper-exporting P-type ATPase	0.938649	0.0166494	17	29.1	Q838Y5
Spermidine/putrescine ABC transporter, ATP-binding protein	0.935763	0.0242974	2	6.9	Q835Z8
Cold shock protein CspC	0.826335	0.0414736	4	80.3	Q833G3
Cellulose biosynthesis cyclic di-GMP-binding regulatory protein BcsB	0.809572	0.0170368	12	23	Q837F4
DUF2187 domain-containing protein	0.75339	0.0432523	3	63.8	Q835R0
Segregation and condensation protein A	0.692021	0.0318054	2	9.1	Q834U4
30S ribosomal protein S15	0.690959	0.00618528	3	39.3	Q82ZJ1
Thioredoxin	0.690918	0.0330177	5	79.8	Q835H2
Uncharacterized protein	0.688846	0.0210995	3	47.7	Q830I5
Guanosine monophosphate reductase	0.656947	0.030067	8	35.1	Q831S1

**Table 3:** Proteins which are uniquely absent in *E. faecalis* when grown in the presence of gliotoxin (5 µg/ml), compared to identical cultures grown without gliotoxin (5 µg/ml). Grown using tryptic soy broth (TSB) media for 30 min in the log phase (0.3–0.4 OD600).

Protein description	Fold Change (log2)	*p*-value	Peptides	Sequence coverage [%]	Protein IDs
Endonuclea_NS_2 domain-containing protein	Absent	N/A	2	17.4	Q835Q6
ABC transporter, ATP-binding/permease protein	Absent	N/A	5	12.3	Q837A1
Uncharacterized protein	Absent	N/A	3	41.7	Q82YV6
Lipase, putative	Absent	N/A	4	15.1	Q82Z80
ATP-dependent DNA helicase RecG	Absent	N/A	4	8.6	Q82ZE7
YitT family protein	Absent	N/A	3	12.4	Q82ZG6
Beta-hydroxyacyl-ACP dehydratase	Absent	N/A	2	27.3	Q833N7
Phosphate transport system permease protein PstA	Absent	N/A	2	7.1	Q834B2
ABC transporter, ATP-binding protein	Absent	N/A	3	23.2	Q835Q5
PTS system, IIA component	Absent	N/A	3	50	Q836T9
DUF2200 domain-containing protein*	Absent	N/A	4	49.1	Q833J9
YlbF family regulator*	Absent	N/A	2	20.1	Q831P6

*This protein exhibited overall decreased abundance > 1.5-fold and was only detectable in 1 of 3 treated samples.

**Table 4:** Proteins which are decreased in abundance in *E. faecalis* when grown in the presence of gliotoxin (5 µg/ml), compared to identical cultures grown without gliotoxin (5 µg/ml). Grown using tryptic soy broth (TSB) media for 30 min in the log phase (0.3–0.4 OD600).

Protein description	Fold Change (log2)	*P*-value	Peptides	Sequence coverage [%]	Protein IDs
30S ribosomal protein S12	− 2.08302	0.0128374	2	25.5	Q839H1
Sodium/dicarboxylate symporter family protein	− 0.929064	0.0460545	11	23.5	Q837T6
Cysteine synthase B, putative	− 0.843203	0.0200332	10	49.8	Q838Z3
Cyclopropane-fatty-acyl-phospholipid synthase	− 0.711893	0.0158166	15	54.9	Q839G6
Magnesium-transporting ATPase, P-type 1	− 0.63794	0.0195624	16	23.7	Q835M5

**Table 5:** Proteins which are uniquely present in *E. faecalis* when grown in the presence of gliotoxin (5 µg/ml), compared to identical cultures grown without gliotoxin (5 µg/ml). Grown using tryptic soy broth (TSB) media for 60 min in the log phase (0.3–0.4 OD600).

Protein description	Fold Change (log2)	*p*-value	Peptides	Sequence coverage [%]	Protein IDs
Pyridine nucleotide-disulphide oxidoreductase	Unique	N/A	11	33.2	H7C719
50S ribosomal protein L33 3	Unique	N/A	2	26.5	P59628
50S ribosomal protein L33 4	Unique	N/A	3	44.9	P59629
Cobalamin synthesis protein/P47K family protein	Unique	N/A	6	25.6	Q82Z69
50S ribosomal protein L28	Unique	N/A	4	48.4	Q82ZE4
UPF0637 protein EF_3078	Unique	N/A	3	26.6	Q82ZH9
Glycosyl transferase, group 2 family protein	Unique	N/A	4	18.7	Q832N3
Na+/H+ antiporter, putative	Unique	N/A	7	11.4	Q834R5
MazG domain-containing protein	Unique	N/A	3	28.3	Q836X0
Tributyrin esterase, putative	Unique	N/A	2	8	Q837P7
Uncharacterized protein	Unique	N/A	7	71.9	H7C700
Transcriptional regulator MraZ	Unique	N/A	4	27.3	O07103
Glycerol kinase	Unique	N/A	24	72.1	O34154
Gluconate kinase, putative	Unique	N/A	6	17	Q82Z43
30S ribosomal protein S14 1	Unique	N/A	1	13.5	Q8KU58
OsmC/Ohr family protein	Unique	N/A	2	19.2	Q82Z71
ATP-dependent DNA helicase RecG	Unique	N/A	7	14.2	Q82ZE7
Serine/threonine transporter SstT	Unique	N/A	4	11	Q82ZN5
Lipoprotein, putative	Unique	N/A	3	13.5	Q82ZP7
Hydrolase, haloacid dehalogenase-like family	Unique	N/A	5	31.3	Q82ZY1
Hydroxyethylthiazole kinase	Unique	N/A	3	13.6	Q830K4
Transcriptional regulator	Unique	N/A	6	37.1	Q830S0
Acetyltransferase, GNAT family	Unique	N/A	4	27.6	Q830S1
DUF2188 domain-containing protein	Unique	N/A	5	33.3	Q833X9
Lipoprotein signal peptidase	Unique	N/A	2	16.1	Q834D8
Sucrose operon repressor ScrR	Unique	N/A	5	30.1	Q834N9
Phosphoenolpyruvate–glycerone phosphotransferase	Unique	N/A	3	18.2	Q835L8
Uncharacterized protein	Unique	N/A	1	22	Q835Q1
ABC transporter, ATP-binding protein	Unique	N/A	4	29.2	Q835Q5
Transcriptional regulator, Cro/CI family	Unique	N/A	8	33.5	Q835Q9
Amino acid ABC transporter, amino acid-binding protein	Unique	N/A	5	28.4	Q836J2
50S ribosomal protein L32-3	Unique	N/A	4	37.3	Q836R0
ABC transporter permease	Unique	N/A	3	13.4	Q837A0
Isopentenyl-diphosphate delta-isomerase	Unique	N/A	4	15	Q837E2
50S ribosomal protein L36*	Unique	N/A	2	60.5	Q839E1
ABC transporter, ATP-binding protein*	Unique	N/A	7	47	Q839U4
Dihydropteroate synthase*	Unique	N/A	4	20.9	Q82Z14
CBS domain protein*	Unique	N/A	8	54.7	Q830P4
4-hydroxy-tetrahydrodipicolinate synthase*	Unique	N/A	10	54.5	Q836D1

*This protein exhibited overall increased abundance > 1.5-fold and was only detectable in 1 of 3 untreated samples.

**Table 6:** Proteins which are increased in abundance in *E. faecalis* when grown in the presence of gliotoxin (5 µg/ml), compared to identical cultures grown without gliotoxin (5 µg/ml). Grown using tryptic soy broth (TSB) media for 60 min in the log phase (0.3–0.4 OD600).

Protein description	Fold Change (log2)	*p*-value	Peptides	Sequence coverage [%]	Protein IDs
Adhesion lipoprotein (adcA-II)	8.49828	0.0020639	17	47.2	Q82Z67
Adhesion lipoprotein (adcABC)	2.61741	0.00538351	8	44.8	Q839U5
Peptide ABC transporter, ATP-binding protein	1.28554	0.00946214	12	70.7	Q82ZF0
5'-nucleotidase family protein	0.948218	0.011422	23	26.9	Q839U0
LemA family protein	0.664767	0.00432472	6	40.7	Q838I0
Phage portal protein	0.64703	0.0486542	16	56	Q835K9
ABC transporter, ATP-binding protein	0.643841	0.0456337	4	35.8	Q833S0
Xanthine phosphoribosyltransferase	0.614899	0.00209845	12	84.5	Q831Y0
Adenine phosphoribosyltransferase	0.575857	0.0317383	10	71.2	Q834G6
Peptide methionine sulfoxide reductase MsrB*	1.6171	N/A	5	51.7	P0DM32

*This protein was found in 2/3 GT treated samples and 1/3 untreated controls which prevented calculation of a P-value.

**Table 7:** Proteins which are uniquely absent in *E. faecalis* when grown in the presence of gliotoxin (5 µg/ml), compared to identical cultures grown without gliotoxin (5 µg/ml). Grown using tryptic soy broth (TSB) media for 60 min in the log phase (0.3–0.4 OD600).

Protein description	Fold Change (log2)	*p*-value	Peptides	Sequence coverage [%]	Protein IDs
Glycosyl hydrolase, family 1	Absent	N/A	9	22.8	Q836T7
Thymidine kinase	Absent	N/A	5	29.9	Q831F5
Probable transcriptional regulatory protein EF_0663	Absent	N/A	3	22.3	Q838A9
Transcriptional regulator, MerR family	Absent	N/A	4	15.3	Q832K1
DNA-binding response regulator	Absent	N/A	5	25.3	Q833S2
Ferrous iron transport protein A	Absent	N/A	3	22.9	Q838H4
Cell division protein FtsL	Absent	N/A	3	40	H7C6Z7
Uncharacterized protein	Absent	N/A	2	19.1	Q833H3
MazG domain-containing protein	Absent	N/A	3	33.6	Q834S7
PTS system, IIA component	Absent	N/A	3	22.6	Q831R2
PC4 domain-containing protein	Absent	N/A	2	48.6	Q82ZD2

**Table 8:** Proteins which are decreased in abundance in *E. faecalis* when grown in the presence of gliotoxin (5 µg/ml), compared to identical cultures grown without gliotoxin (5 µg/ml). Grown using tryptic soy broth (TSB) media for 60 min in the log phase (0.3–0.4 OD600).

Protein description	Fold Change (log2)	*p*-value	Peptides	Sequence coverage [%]	Protein IDs
Sodium/dicarboxylate symporter family protein	− 0.6585	0.008642	11	26.8	Q837T6
Mn^2+^ /Fe^2+^ transporter, NRAMP family	− 0.61846	0.026495	2	5.6	Q836Q1
PTS system mannose-specific EIIAB component	− 0.60834	0.007484	22	78.8	Q839X9
PTS system, mannose-specific IIC component	− 0.6067	0.018615	2	12.4	Q839X8

**Table 9:** Proteins which are uniquely present in *E. faecalis* when grown in the presence of gliotoxin (5 µg/ml), compared to identical cultures grown without gliotoxin (5 µg/ml). Grown using tryptic soy broth (TSB) media for 180 min in the log phase (0.3–0.4 OD600).

Protein description	Fold Change (log2)	*p*-value	Peptides	Sequence coverage [%]	Protein IDs
ABC transporter, ATP-binding protein	Unique	N/A	4	24.1	Q833B4
UPF0316 protein EF_1609	Unique	N/A	4	22.4	Q834N5
Uracil-DNA glycosylase	Unique	N/A	7	42.9	Q836Z5
ABC transporter, ATP-binding protein	Unique	N/A	7	38.7	Q839U4
50S ribosomal protein L33 4	Unique	N/A	2	40.8	P59629
30S ribosomal protein S14 2	Unique	N/A	3	39.3	Q82Z70
Lipoprotein, putative	Unique	N/A	3	19.6	Q82ZP7
DUF2829 domain-containing protein	Unique	N/A	4	57.1	Q833Z9
MutT/nudix family protein	Unique	N/A	4	23.7	Q834Q4
UPF0291 protein EF_1580	Unique	N/A	4	37.5	Q834Q9
Histidine kinase	Unique	N/A	6	12.7	Q835W1
Amino acid ABC transporter, ATP-binding protein	Unique	N/A	7	41.2	Q837N1
Transcriptional regulator, ArsR family	Unique	N/A	3	26.1	Q839Q2
Adhesion lipoprotein (AdcABC)*	Unique	N/A	9	44.8	Q839U5
Uncharacterized protein*	Unique	N/A	2	32.2	Q835Q1
Adhesion lipoprotein (AdcA-II)*	Unique	N/A	22	51.9	Q82Z67

*This protein exhibited overall increased abundance > 1.5-fold and was only detectable in 1 of 3 untreated samples.

**Table 10:** Proteins which are increased in abundance in *E. faecalis* when grown in the presence of gliotoxin (5 µg/ml), compared to identical cultures grown without gliotoxin (5 µg/ml). Grown using tryptic soy broth (TSB) media for 180 min in the log phase (0.3–0.4 OD600).

Protein description	Fold Change (log2)	*p*-value	Peptides	Sequence coverage [%]	Protein IDs
Pyridoxal phosphate homeostasis protein	1.84387	0.0140765	8	64	Q836V5
Transcriptional regulator, GntR family	0.915665	0.0140919	17	68.2	Q833B3
Uncharacterized protein	0.854289	0.0111249	7	85.6	Q833L4
Methylglyoxal synthase	0.778122	0.0358049	5	52.1	Q837A4
Cysteine synthase	0.748689	0.0245636	18	77.1	Q834Q6
D-isomer specific 2-hydroxyacid dehydrogenase family protein	0.690603	0.00288734	8	39.1	Q82ZZ6
Oxidoreductase, short chain dehydrogenase/reductase family	0.64496	0.0218784	13	47.8	Q839T0
Ribosomal RNA small subunit methyltransferase I	0.588148	0.0470489	10	38.3	Q830M1

**Table 11:** Proteins which are uniquely absent in *E. faecalis* when grown in the presence of gliotoxin (5 µg/ml), compared to identical cultures grown without gliotoxin (5 µg/ml). Grown using tryptic soy broth (TSB) media for 180 min in the log phase (0.3–0.4 OD600).

Protein description	Fold Change (log2)	*p*-value	Peptides	Sequence coverage [%]	Protein IDs
50S ribosomal protein L33 3	Absent	N/A	2	26.5	P59628
PTS system, beta-glucoside-specific IIABC component	Absent	N/A	5	15.2	Q831B4
6-aminohexanoate-cyclic-dimer hydrolase, putative	Absent	N/A	10	19.1	Q836S5
Prephenate dehydrogenase	Absent	N/A	7	23.4	H7C6X1
Mga domain-containing protein	Absent	N/A	6	15.6	Q82ZN7
Protease synthase and sporulation negative regulatory protein pai 1	Absent	N/A	2	11.7	Q82ZP9
Transcriptional antiterminator, bglG family	Absent	N/A	3	7	Q82ZT1
FolC family protein	Absent	N/A	6	19.3	Q82ZW9
DNA polymerase III, delta prime subunit	Absent	N/A	3	8.9	Q830L8
Acetyltransferase, GNAT family	Absent	N/A	3	21.3	Q830S1
Diacylglycerol kinase catalytic domain protein	Absent	N/A	3	12.9	Q830V8
ABC transporter, permease protein	Absent	N/A	3	16.7	Q831K7
ComE operon protein 2, putative	Absent	N/A	4	29.3	Q831Q2
Uncharacterized protein	Absent	N/A	1	25.5	Q831U5
YxeA family protein	Absent	N/A	3	26.5	Q832L5
tRNA-specific adenosine deaminase	Absent	N/A	2	16.2	Q832M0
Probable dual-specificity RNA methyltransferase RlmN	Absent	N/A	9	34.5	Q833B6
Putative 3-methyladenine DNA glycosylase	Absent	N/A	4	19.2	Q833H5
YitT family protein	Absent	N/A	5	21.2	Q833I6
Uncharacterized protein	Absent	N/A	3	63.3	Q833K1
Glycerol uptake facilitator protein	Absent	N/A	2	9.8	Q833L8
Phosphate transport system permease protein PstA	Absent	N/A	2	7.1	Q834B2
Bacteriocin-protection, YdeI or OmpD-Associated	Absent	N/A	2	10.5	Q834C8
Cold-shock domain family protein	Absent	N/A	2	63.2	Q834D5
Metal-independent alpha-mannosidase	Absent	N/A	10	33.8	Q834E7
Glyoxylase family protein	Absent	N/A	5	18.8	Q834I3
V-type ATP synthase subunit D	Absent	N/A	4	32.7	Q834X7
Exodeoxyribonuclease 7 small subunit	Absent	N/A	2	46.1	Q836W5
Pyrroline-5-carboxylate reductase	Absent	N/A	3	14.8	Q836Y3
3-demethylubiquinone-9 3-methyltransferase	Absent	N/A	2	12.1	Q837B9
UPF0178 protein EF_0842	Absent	N/A	5	45.3	Q837J5
DUF1189 domain-containing protein	Absent	N/A	2	8.2	Q838I5
Putative N-acetylmannosamine-6-phosphate 2-epimerase	Absent	N/A	3	18.1	Q839T3
4-diphosphocytidyl-2-C-methyl-D-erythritol kinase	Absent	N/A	4	15.2	Q839U9
PTS system, IIB component*	Absent	N/A	8	50	Q82ZC7

*This protein exhibited overall decreased abundance > 1.5-fold and was only detectable in 1 of 3 treated samples.

**Table 12:** Proteins which are decreased in abundance in *E. faecalis* when grown in the presence of gliotoxin (5 µg/ml), compared to identical cultures grown without gliotoxin (5 µg/ml). Grown using tryptic soy broth (TSB) media for 180 min in the log phase (0.3–0.4 OD600).

Protein description	Fold Change (log2)	*p*-value	Peptides	Sequence coverage [%]	Protein IDs
Glycosyl hydrolase, family 1	− 3.88117	0.0106769	13	34.1	Q831B5
Glycerol kinase	− 2.9383	0.00501984	24	64.1	O34154
Alpha-glycerophosphate oxidase	− 2.89753	0.0117899	33	68.1	Q833L7
Glycosyl hydrolase, family 1	− 2.76907	0.012451	15	39.9	Q839A6
PTS system, IIBC components	− 2.73649	0.0463181	7	16.2	Q832L3
ABC transporter, substrate-binding protein	− 1.82212	0.0241417	19	49.8	Q832K5
Ornithine carbamoyltransferase, catabolic	− 1.73602	0.00170647	17	67.6	Q839Q5
30S ribosomal protein S15	− 1.64109	0.0088054	6	56.2	Q82ZJ1
Hydrolase, haloacid dehalogenase-like family	− 1.4352	0.0409972	6	48.6	Q82ZA8
Acyl carrier protein	− 1.34991	0.0196974	3	88	Q830B0
Biotin carboxyl carrier protein of acetyl-CoA carboxylase	− 1.32096	0.0104951	4	32.1	Q830B2
Acyl carrier protein	− 1.16376	0.0267896	3	55.7	Q82ZE9
Arginine deiminase	− 1.09113	0.00625365	25	74.3	Q93K67
Mn^2+^/Fe^2+^ transporter, NRAMP family	− 1.04732	0.0290044	3	11.1	Q836Q1
Carbamate kinase 1	− 1.02868	0.0133442	14	66.8	P0A2X7
Citrate lyase subunit beta	− 0.941302	0.017623	8	26.4	Q82YW2
Sodium/dicarboxylate symporter family protein	− 0.814864	0.0266616	11	29.2	Q837T6

Three ribosomal proteins are either uniquely present or increased in abundance following GT addition, and two Zn^2+^ uptake membrane proteins (AdcA and AdcC of the Gram positive AdcABC system), out of 32 proteins in this dataset, were significantly increased in abundance (log_2_-fold 2.4–7.28) at 30 min (Tables 1, 2; Supplementary Fig. 9). This is the first demonstration that GT affects bacterial ribosomal protein presence, as has been predicted^39^. Increased Zn^2+^ uptake transporter abundance clearly suggests that intracellular Zn^2+^ may be complexed by GT/DTG resulting in a limiting intracellular environment. Our observations complement previous work which revealed significantly increased *adcABC* and *adcA-II* gene expression following exposure to 5 µM TPEN^46^. Moreover, it is in complete accordance with our data which shows that Zn^2+^ relieves GT-mediated growth inhibition and that GT/DTG is a Zn^2+^ chelator. It is further conceivable that DTG may eject Zn^2+^ from intracellular proteins resulting in inactivity and possibly denaturation. The increased abundance of the Zn^2+^ uptake membrane proteins was also evident at T = 60 and they were uniquely detected at T = 180 min post-GT addition (Tables 5, 6, 9, 10).

Our hypothesis that GT-mediated zinc depletion (reduced bioavailability) was primarily responsible for quantitative proteomic alterations, was investigated by assessment if Zn co-addition (200 µM) for 30 min with GT would reverse the observed GT-induced proteomic alterations. This chemo-complementation approach revealed that 1 of 16 proteins uniquely detected by GT addition only exhibited reversed abundance in the presence of GT and Zn, of the 12 proteins uniquely absent with GT, 3 were decreased in abundance in the presence of Zn while 1 (Q834B2) was uniquely detected in the Zn supplemented samples (Supplementary Tables 4 and 5). After 30 min exposure to GT (5 µg/ml) and Zn (200 µM), there was a minor and insignificant increase in AdcA, AdcC, and AdcA-II when compared to GT (5 µg/ml) only. This data indicates that 30 min may be too short a time frame to see a complete disappearance of the AdcABC system proteins. After 60 min, AdcA was decreased in abundance (− 1.5 log2 fold change), AdcC also exhibited decreased abundance (− 1.3 log2 fold change) (although not statistically significant (*p* < 0.5)) and AdcA-II was also insignificantly decreased in abundance (*p* < 0.2; − 1.3 log2 fold change) in the presence of GT (5 µg/ml) and Zn (400 µM), when compared to GT (5 µg/ml) only (Supplementary Table 6). In combination, these data indicate that addition of Zn (400 µM) relieves some of the effects of GT (5 µg/ml), but was either insufficient to result in a rapid decrease in the requirement for the Zur-regulated zinc uptake system or 60 min incubation was sub-optimal for this comparative study. Hence, future work will focus on evaluating and defining the optimal conditions of zinc reversal of significantly elevated AdcABC and Adc-II uptake systems in *E. faecalis*.

However, the increased abundance or unique presence of *E. faecalis* zinc import proteins (AdcA and C) in the presence of GT at all timepoints is in complete accordance with the independent demonstration that disruption of orthologous genes encoding AdcA or AdcB and AdcC, components of the zinc uptake system in *Streptococcus mutans*, caused severe growth inability under zinc-deplete conditions^53^. Furthermore, it was demonstrated that the *S. mutans* ΔadcBC mutant exhibited a severe colonisation defect in a rat infection model system^53^. In a further study in *E. faecalis*, it has been elegantly shown that AdcABC and adhesion lipoprotein AdcA-II function in a cooperative manner to ensure Zn homeostasis. Deletion of system components resulted in zinc-associated growth defects and increased sensitivity to antibiotics which target the bacterial cell wall^54^. These authors also showed that bacterial virulence was attenuated in zinc transporter deletion mutants and propose interference with high affinity zinc importers as a potential therapeutic strategy to combat *E. faecalis* infection^54^. In combination, these deletion studies and associated identification of attenuated virulence in the absence of zinc uptake systems underpin the strategy and utility of using GT/DTG couple as a means of identifying new antibacterial drug targets^38^. Interestingly, an *E. faecalis* pyridine nucleotide-disulphide oxidoreductase (PNDO)(H7C719) was uniquely detected in the presence of GT (Table 1 and 5) at T = 30 and 60 min exposure. To our knowledge, this is a protein of unknown function in *E. faecalis* and its presence is suggestive of altered redox homeostasis upon GT exposure. It is also relevant that the self-protection enzyme gliotoxin oxidoreductase GliT in *A. fumigatus* converts DTG to GT^22,23^ and is also classified as a PNDO-type enzyme. Future work will involve targeting this bacterial PNDO for deletion and mutant characterisation.

The unique or elevated abundance of specific ribosomal proteins ((T30 = 50S ribosomal protein L33 4 (P59629), 30S ribosomal protein S14 1 (Q8KU58), and 30S ribosomal protein S15 (Q82ZJ1); T60 = 50S ribosomal protein L33 4 (P59629), 50S ribosomal protein L28 (Q82ZE4), 30S ribosomal protein S14 1 (Q8KU58); T180 = 50S ribosomal protein L33 4 (P59629), 30S ribosomal protein S14 2 (Q82Z70); (plus uniquely absent 50S ribosomal protein L33 3 (P59628)) was also observed (Supplementary Fig. 10 and Supplemental Table 1). These changes in ribosomal protein abundance were indicative of a shift towards non-zinc binding paralogs upon GT addition, which is in accordance with the proposal of Danchin that the prokaryotic ribosome may act as a zinc store^39^. Thus, when GT is added to *E. faecalis*, we hypothesise that resultant zinc depletion would lead to zinc release from ribosomes and consequent increased presence of selected zinc-free ribosomal protein paralogs. Future RNAseq work will further investigate this GT-induced phenomenon in *E. faecalis*, as has been deployed in *Mycobacterium smegmatis*^55^. In the work of Dow et al., non-zinc-binding paralogs were identified as functional replacements for Zn^2+^-dependent paralogs and were involved in the transcriptomic response to Zn^2+^-limitation.

GT uptake and predicted conversion to DTG by intracellular reduction by GSH, L-Cys or other reductants would likely lead to dissipation of these metabolites and increased biosynthesis of same. In accordance with this prediction, a cysteine synthase (Q834Q6) was detected as significantly increased in abundance (*p* < 0.025) at T = 180 min, with high confidence given that 77% sequence coverage was evident (Tables 9,10). This enzyme may result in either L-Cys or ultimately GSH formation in *E. faecalis*. Importantly, this proteomic observation aligns with the detection of higher (+ 38%) total intracellular thiol concentration (0.156 nmol per mg cells) following GT addition to *E. faecalis*. Detection of a putative cysteine synthase B (Q838Z3) as significantly decreased in abundance (*p* < 0.02) at T = 30 min requires future analysis (Tables 3, 4).

Detection of a cobalamin synthesis protein (Q82Z69) at T = 30 and 60 only post-GT addition (Tables 1, 2, 5, 6) suggests that vitamin B_12_ biosynthesis is increased in *E. faecalis* due to GT presence. Allied to this is the increased abundance of Met sulphoxide reductase MsrB (P0DM32) which was identified in 2/3 GT treated samples versus 1/3 controls therefore precluding the calculation of a* p*-value (Tables 5, 6). This enzyme has been shown to contribute to *E. faecalis* response to oxidative stress, by L-Met regeneration, and virulence^56^. The detection of both these enzymes, along with significantly increased abundance (*p* < 0.03) of thioredoxin (Table 1, 2) suggests that the GT effect on *E. faecalis* also involves disrupted L-Met oxidation, redox homeostasis and possibly Methyl/Met cycle due to SAM requirements. Future work will investigate the altered abundance of enzymes involved in L-Met metabolism by targeted deletion studies, since few data is currently available on these phenotypes.

Our quantitative proteomic analyses also revealed absence or significantly reduced abundance of components of the *E. faecalis* PTS (phosphoenolpyruvate (PEP):carbohydrate phosphotransferase system), mainly PTS system: IIA component, mannose-specific EIIAB and IIC components between 30 and 180 min post-GT addition, though one protein in this system, PTS System, beta-glycoside-specific IIABC component was uniquely present with GT (Q831B4) (Tables 1, 3, 4, 7, 8, 11, 12). This system has recently been shown to repress ribosomal biosynthesis and to increase the sensitivity of *E. faecalis* to gentamycin. Overexpression of PTS also increased the sensitivity of *E. faecalis* to the antibiotic, daptomycin^57^. Interestingly, alkaline stress caused reduced abundance of PTS system components in E*. faecalis* V583 as determined by proteomic analysis^58^. Our observation of decreased abundance of PTS system components, in response to GT presence is in accordance with these studies which suggests that deactivation of the PTS system reduces the impact of antibacterial compounds (e.g., daptomycin, GT or gentamycin) or conditions (alkaline stress).

Through proteomic analysis we show that GT may affect intracellular metal ion homeostasis, possibly by interference with intracellular zinc pools in *E. faecalis*. Proteins associated with key pathways, several of which are controlled by the zinc-dependent Zur transcription factor^46^, were altered in abundance when *E. faecalis* was exposed to GT. This metallo-centric model implicates DTG as the active form of GT and supplants the conventional perspective of altered redox homeostasis due to GT presence as the sole factor responsible for the antimicrobial activity of GT/DTG. In conclusion, this work illustrates the GT/DTG couple potential for providing new ways to combat the growing antibiotic resistance threat as well as yielding new mechanistic approaches for treating antibiotic resistant infections^38^.

In summary, DTG causes Zn^2+^ and Cu^2+^ to specifically dissociate from PAR in a concentration-dependent manner. DTG can specifically strip or remove Fe^3+^ from low and high sensitivity Fe^3+^ chelates in a concentration-dependent manner. Notably, this interaction is also time-dependent. The reducing agent DTT and antioxidant GSH had no effect on fluorescent chelate:Fe^3+^ stability, implicating chelation as the mechanism of Fe^3+^ removal from this chelate by DTG. When exposed to GT (5 µg/ml) *E. faecalis* showed significant and specific proteome alterations, many of which are linked to the occurrence Zn^2+^-limiting or bioavailability conditions; and for the first time imply that GT/DTG induce zinc starvation within *E. faecalis*. DTG may chelate free Zn^2+^ and eject it from intracellular metalloproteins, thereby causing growth inhibition. During manuscript revision a report emerged which also addressed the role of gliotoxin as an anti-bacterial agent^59^.

## Methods

### Bacterial growth inhibition assays

Single colonies of *E. faecalis* (ATCC 19433), *E. faecium* (ATCC 19434), *K. pneumoniae* (ATCC 70063), and *A. baumannii* (ATCC 19606) were used to inoculate tryptic soy broth (TSB) (Sigma-Aldrich, cat no. 22092-500G). These cultures were incubated overnight (37°C; 170 rpm). The OD600 nm of the cultures were determined and aliquots of each culture were diluted to OD600 nm = 0.2 using TSB. Cultures were added to wells (100 µl/well) of 96-well plates, in triplicate, containing 100 µl TSB supplemented with GT (0–120 µM) and Zn, Cu, or Mn (0, 20, 200, or 400 µM), incubated (37°C; Static; 18 h) and OD600 nm determined using a BioTek Synergy HT 96-well plate reader. Following GT exposure (GT: 0–60 µM), iron supplementation (0, 10, 100 and 200 µM) was carried out for *E. faecalis* only. TSB has been shown via ICP-AES^41^ to basally contain: Zn: 0.6 ppm = 9.177 µM; Cu: < 0.1 ppm =  < 1.5737 µM and Fe: 0.3 ppm = 5.372 µM. Data analysis was performed using GraphPad Prism 9.5.0 via two-way ANOVA.

### Zn determination by zinquin method

*E. faecalis* (ATCC 19433) was grown overnight in TSB (37 °C 170 rpm). These cultures were used to inoculate fresh TSB to an OD600 of 0.05 until reaching an OD600 of 0.3–0.4. The cultures were divided into even aliquots and spiked with GT (5 µg/ml final) or MeOH. The cultures were returned to the incubator (37 °C 170 rpm) and 15 ml aliquots were taken after 5, 15, and 30 min. The assay was also performed for 60 min. Aliquots were centrifuged 4500 g at 4 °C for 10 min and the supernatants discarded. Pellets were washed extensively in PBS and transferred to 1.5 ml tubes. Samples were centrifuged 15,000 g for 10 min at 4 °C. After weighing pellets, 400 µl PBS (0.03% (w/v) albumin from chicken egg white) was added to each sample and vortexed into solution followed by sonication on ice (6 times MS72 probe, power ≤ 20%, cycle 6) and transfer to black 96 well plates (200 µl per well) for addition of 3.5 µl Zinquin in DMSO (12 mM stock). After pipette-mixing and incubation (37 °C in the dark for 40 min.), Zn levels were calculated by comparison to a Zn standard curve. All dilutions were done in PBS (0.03% albumin from chicken egg white). Fluorescence detection: excitation: 360/40 nm, emission: 460/40 nm, Gain: 65.

### GT versus TPEN bacterial growth Inhibition assays

*E. faecalis* was added to wells (100 µl/well) of 96-well plates, in triplicate, containing 100 µl TSB supplemented with GT (0–120 µM) or TPEN (0–120 µM) and ZnSO_4_ (0, 20, 200, or 400 µM), incubated (37 °C; static; 18 h) and OD600 nm determined using a BioTek Synergy HT 96-well plate reader. Data analysis was performed using GraphPad Prism 9.5.0 via two-way ANOVA.

### GT-augmented vancomycin bacterial growth inhibition assays, with and without Zn

*E. faecalis* was added to wells (100 µl/well) of 96-well plates, in triplicate, containing 100 µl TSB supplemented with GT (0–20 µg/ml) and vancomycin (0–64 µg/ml) in varied concentrations. The wells were then inoculated with 100 µl *E. faecalis* (0.2 OD 600 nm). Identical plates were then prepared using wells supplemented with ZnSO_4_ (200 µM final concentration). The plates were incubated (37 °C; static; 18 h). The wells were imaged using a camera attached to a microscope (Olympus SZX16 microscope with Olympus SDF PLAPO 2XPFC camera attachment) using cellSens Standard software. The wells were homogenised using a multichannel pipette OD600 nm determined using a BioTek Synergy HT 96-well plate reader.

### Gliotoxin x vancomycin biofilm evaluation by crystal violet assay

*E. faecalis* cultures (0.2 OD600) were added to 96-well plates, 100 µl/well, containing 100 µl TSB supplemented with GT (0–10 µg/ml) and vancomycin (4 µg/ml) followed by incubation (37 °C; Static), 18 h and OD600 determined using a BioTek Synergy HT 96-well plate reader. The media was removed from the wells, washed with deionised H_2_O then dried. Crystal violet (0.1% (w/v)) was added to each well, incubated for 10 min at room temperature, discarded, the wells washed with deionised H_2_O and then dried. Acetic acid (30% (v/v)) was added to each well and allowed to incubate for 10 min at room temperature. The wells were pipette-mixed then read at 595 nm using a BioTek Synergy HT 96-well plate reader.

### GT time-kill assay

Three colonies of *E. faecalis* were used to inoculate TSB followed by incubation overnight (37 °C; 170 rpm). The OD600 of the cultures were measured and an aliquot of each culture was taken and diluted to 5 × 10^5^ CFU/ml using TSB. These cultures were divided into aliquots and spiked with GT in MeOH (0, 1.875, 3.75 and 7.5 µM) and further divided into aliquots for each time point in falcon tubes. After incubation (37 °C; 170 rpm), culture growth was analysed at 0, 2, 4, 8, and 24 h whereby at each time point the cultures were serially diluted in TSB and streaked on TSB agar. The plates were grown (37 °C; Static) and counted after 24 h.

### Assessment of DTG metal chelation capacity by 4-(2-pyridylazo)resorcinol (PAR) assay

A master stock of 4-(2-pyridylazo)resorcinol (PAR) (1.5 mM)^60^ was prepared in PBS pH 7.4. A working stock of PAR was made up in PBS pH 7.4 (22.22 µM). PAR (22.22 µM; 180 µl) was transferred to the wells of a 96-well plate. 10 µl of 200 µM metal (Zn, Cu) was added to the appropriate wells. The plate was allowed to incubate in the dark for 2 min and 10 µl of DTG or N,N,N′,N′-Tetrakis(2-pyridylmethyl)ethylenediamine (TPEN) (0–120 µM final) was added to the appropriate wells and read using a BioTek Synergy HT 96-well plate reader with spectrum scan 300–600 nm.

### Determination of DTG and TPEN Zn pKd

This methodology was adapted from that of Kocyła et al., with the 10 h incubation being done in plastic cuvettes instead of glass^60^.

### Assessment of DTG and related thiol compound reductant activity by Siderotec™ assays

The Siderotec™ total and Siderotec-HiSens™ tests were performed as per manufacturer’s instructions (Accuplex Diagnostics Limited, Ireland). In brief, these tests deploy chromophoric and fluorimetric compounds, respectively, to detect Fe^3+^ chelator or reductant activity^61^, Supplemental information and Supplementary Figs. 6 and 7.

### High resolution mass spectrometry analysis of GT metal chelates

Tris(2-carboxyethyl)phosphine hydrochloride (TCEP)-reduced GT (DTG; 304.5 μM final) in 50%(v/v) MeOH was spiked with ZnSO_4_·7H_2_O, CuCl_2_, or FeSO_4_·7H_2_O respectively to achieve 1 and threefold molar equivalents of metals to DTG (DTG; 300 μM final). Formic acid was added to the samples (0.1%(v/v) final). Samples were filtered (0.22 μm spin filters) and directly injected onto a Thermo Q-Exactive Mass spectrometer (5 μl/min). Assessment of DTG-metal complex formation was evaluated by both positive and negative ESI mode at 70,000 resolution with full MS scan (*m/z* 66.7–1000.00). The following settings were used in the analysis: spray voltage (negative mode: 3.6 kV, positive mode 4 kV), capillary temperature 320 °C.

### Quantitative proteomic analysis of *E. faecalis* response to GT exposure

Three biological replicate *E. faecalis* cultures were grown overnight in TSB. These cultures were then used to inoculate warm TSB to an OD600 nm 0.05 and grown (37°C; 170 rpm) until log phase (OD600 nm 0.3). At this stage, each culture was divided into two equal aliquots, one of each was spiked with GT (5 µg/ml final) while the other was spiked with the same volume of MeOH (solvent control). The cultures were returned to the incubator (37°C; 170 rpm) with aliquots taken at T = 30, 60 and 180 min post spiking. Cell pellets were collected and stored at -20°C until required. Pellets were prepared using a modified FASP protocol^62^ as follows: pellets were lysed via sonication whereby 25 mg bacteria was resuspended in 150 μl of 1% (w/v) SDS/500 mM ammonium bicarbonate pH 8.3. Lysed samples were heated at 95°C for 5 min. The samples were then centrifuged at 16,000 g for 5 min, supernatants were collected, and protein content quantified using Qubit protein assay. A 20 µg protein sample was taken from each and transferred to 30 kDa molecular weight cut-off (MWCO) centrifugal filters (Sartorius, cat no. VN01H22). Each sample was brought up to 200 µl with 8 M urea, 500 mM ammonium bicarbonate. The samples were vortexed then centrifuged at 14,000 g for 20 min. 500 mM ammonium bicarbonate, 8 M urea pH 8.3 (200 µl) was added to each filter. The samples were vortexed, then centrifuged at 14,000 g for 20 min and 100 μl 5 mM TCEP in 8M urea, 500 mM ammonium bicarbonate pH 8.3 was added to each sample filter. The samples were vortexed and incubated at room temperature for 20 min. 500 mM IAA (3 µl) was added to each sample (15 mM final) and samples were vortexed and incubated in the dark at room temperature for 20 min. The samples were centrifuged at 14,000 g for 20 min. 100 μl of 8 M urea, 500 mM ammonium bicarbonate pH 8.3 was added to each filter. The samples were vortexed then centrifuged at 14,000 g for 20 min. Another 100 μl of 8 M urea, 500 mM ammonium bicarbonate was added to each filter. The samples were vortexed then centrifuged at 14,000 g for 20 min. Two sequential washes with 100 μl of 500 mM ammonium bicarbonate pH 8.3 was carried out by addition to each filter, samples vortexed, centrifuged at 14,000 g for 20 min. The filters were then transferred to fresh tubes. 120 μl of 500 mM ammonium bicarbonate, 1 µl ProteaseMax (1% w/v) and 5 μl trypsin (400 ng/μl) was added to each filter followed by a brief vortex. The samples were wrapped in parafilm and placed in a humid chamber at 37°C for 18 h followed by centrifugation 14,000 g for 20 min. 500 mM ammonium bicarbonate pH 8.3 (50 μl) was added to each filter, samples were then centrifuged 14,000 g for 20 min and a 60 μl aliquot was taken from each filtrate. 12 μl of resuspension buffer (2% (v/v) TFA, 20% (v/v) in deionised H_2_O) was added to each aliquot, samples were vortexed and stored at − 20 °C until mass spectrometry analysis. Peptide samples were analysed using a Thermo Fisher Q-Exactive mass spectrometer coupled with a Dionex RSLCnano. LC gradients ran from 4 to 40% B (0.1%(v/v) trifluoroacetic acid in acetonitrile) over 2 h 13 min, and data was collected using a Top15 method for MS/MS scans. Comparative proteome abundance and data analysis was performed using MaxQuant software, with Andromeda used for database searching and Perseus used to organise the data^11,31^.

### Supplementary Information

Supplementary Information.

## Data Availability

All data generated or analysed during this study are included in this published article (and its Supplementary Information files). The proteomic datasets generated during and analysed during the current study are available in the *MassIVE* repository, https://doi.org/doi:10.25345/C5Z892R21. All remaining datasets generated and analysed during the current study are available from the corresponding author on reasonable request.
