# Supplementary material for: Gliotoxin-mediated bacterial growth inhibition is caused by specific metal ion depletion
Source: Sci Rep. 2023 Sep 27;13:16156. doi: 10.1038/s41598-023-43300-w (PMC10533825; doi:10.1038/s41598-023-43300-w)
Supplement: Supplementary file 1 — Supplementary Information. [file 41598_2023_43300_MOESM1_ESM.docx]

**Gliotoxin-mediated bacterial growth inhibition is caused by specific metal ion depletion.**

Shane G. Downes^1^, Rebecca A. Owens^1^, Kieran Walshe^2^, David A. Fitzpatrick^1^, Amber Dorey^3^, Gary W. Jones^4,*^ and Sean Doyle^1,*^.

^1^ Department of Biology, Maynooth University, Co. Kildare, Ireland.

^2^ Accuplex Diagnostics Ltd, Co. Kildare, Ireland.

^3^ Molecular Parasitology Laboratory, Centre for One Health and Ryan Institute, School of Natural Sciences, University of Galway, H91 DK59 Galway, Ireland.

^4^ Centre for Biomedical Science Research, School of Health, Leeds-Beckett University, Leeds, UK.

*** Joint Corresponding authors:**

Professor Sean Doyle, Department of Biology, Maynooth University, Maynooth, Co. Kildare, Ireland.

Professor Gary W. Jones, Centre for Biomedical Science Research, School of Health, Leeds-Beckett University, Leeds, UK.

Email: sean.doyle @mu.ie gary.jones@leedsbeckett.ac.uk

Tel : +353-1-7083858 +44-[113-812 3764](tel:+44%20(0)113%20812%203764)

**Key words:** Antimicrobial resistance; *Enterococcus*; ESKAPE pathogens; zinc; quantitative proteomics.

**Supplementary Figure 1.** Iron supplementation does not relieve GT-mediated growth inhibition of *E. faecalis*. *E. faecalis* growth in the presence of (GT; 0-60 µM) supplemented with Fe (0, 10, 100, or 200 µM).

**B.**

**A.**

**C.**

**Supplementary Figure 2.** Gliotoxin exposure does not change the total intracellular Zn content of *E. faecalis*. Lysates of *E. faecalis* treated with gliotoxin (5 µg/ml) for **a.** 5 minutes, **b.** 15 minutes, and **c.** 30 minutes showed no significant change in total intracellular Zn content as measured by zinquin.

**Supplementary Figure 3.** GT bactericidal activity a time-kill assay was performed using 5 x 10^5^ CFU/ml *E. faecalis* and tested at GT (0 – 7.5 µM). The observed bactericidal effect occurred at this GT concentration due to the requirement for a lower CFU/ml which enabled assay quantification and avoided overgrowth.

**Supplementary Figure 4.** Relief of GT-mediated growth inhibition of *E. faecalis* by Zn^2+^ addition (0 - 200 µM).

**Supplementary Figure 5.** Combinatorial treatment of *E. faecalis* with gliotoxin (5 µg/ml) and vancomycin (4 µg/ml) decreased biofilm levels in a statistically insignificant manner (P>0.05). Biofilm was reduced by up to 23.9% when treated with 1.25 µg/ml gliotoxin. Treatment with gliotoxin (0.625 – 10 µg/ml) showed the largest decrease in detected biofilm (19.5 – 23.9%).

**SideroTec-Total Assay^TM^.** The SideroTec-Total Assay^TM^ is a colorimetric test for use in the detection of siderophores secreted by bacteria or fungi, or for the assessment of synthetic iron chelators. The test can be used with liquid culture either directly or following filtration to remove microorganisms. The test may also be used with other aqueous or solvent based liquids. The chromogenic reagent was prepared just before use whereby Catalyst (R2) was diluted 1:10 in the chromogenic reagent (R1). The volume of R1 and R2 solutions depended on the number of samples to be tested and whether standard or controls are used. Generally, R2 (1.2 ml) was added to R1 (12 ml)). For quantitative assessment, samples and standards (100 μl each) were added to the appropriate wells, in duplicate. Pre-mixed R1 reagent/R2 was then added (100 μl/well) using an 8-channel pipette, incubated for 10 min at (37 ^0^C) and wells were read on a microplate reader at 620 nm.

**Interpretation of results.** For quantitative interpretation, results were obtained by reading the microplate on a microplate reader. Results can be determined by plotting 1/OD vs siderophore concentration (µM) to give a linear standard (**Supplementary Figure 1**). Sample concentration was then determined from the graph using the equation of the trendline plotted (y = mx + c).

**Supplementary Figure 6.** Exemplar SideroTec-Total Assay^TM^ calibration curve (Plot of 1/OD600 nm versus µM concentration). **Sensitivity:** The sensitivity of the test using Deferoxamine as reference material is 1.5 µM. Higher sensitivity may be achieved with other siderophores.

**SideroTec-HiSens Assay^TM^.** The SideroTec-HiSens Assay^TM^ is a fluorometric test for use in the detection of siderophores and other iron chelation compounds that may be present in the low-high nanomolar range. The SideroTec-HiSens Assay^TM^ can be used for direct detection of iron chelation molecules in aqueous based liquids or in selected organic solvents. The assay uses an iron-detector complex to detect iron chelation molecules such as siderophores or other synthetic iron chelators. In the presence of an iron chelator, iron will be removed from the complex resulting in an increase in fluorescence signal. Where an iron chelator is present in a sample a fluorescent signal will be generated that will depend on both the amount of chelator present but also on the affinity of the iron chelator. The assay sensitivity can be varied depending on the fluorescent reader settings used and the recommended GAIN is 75. Increasing the reader GAIN enables approximately 10-fold increase in sensitivity.

**Standard Preparation: Range 1: 0-10,000 ng/ml**: Standards were prepared by serial dilution of the standard supplied in the test kit using diluent reagent. The standard was prepared by serial dilution of the 10000 ng/ml (15 µM) standard, in duplicate. **Range 0-1,000 ng/ml:** The standards supplied with the kit were first diluted 1:10 using the diluent provided by diluting 100 µl of the test kit standard in 900 µl of diluent to give a top standard on 1000 ng/ml. This was then serially diluted using the test kit diluent.

For quantitative assessment, samples and standards (100 µl each) were added to the appropriate wells, in duplicate. Ready-to-use fluorescent detector reagent (100 µl/well) was then added to each well using an 8-channel pipette. After 10 min at 37^0^C, plates were read on a Biotek Synergy platereader at excitation λ 360 nm and emission λ 460 nm.

**Interpretation of results.** For quantitative interpretation, results should be obtained by reading the microplate on a microplate reader. A standard curve should be prepared by plotting Relative Fluorescent Units (RFU) generated by each standard versus siderophore concentration (**Supplementary Figure 2**). Background fluorescence (zero standard or matrix only) was subtracted before generation of the standard curve.

**Supplementary Figure 7**. Exemplar SideroTec-HiSens Assay^TM^ calibration curve. **Sensitivity:** The sensitivity of the test using Deferoxamine as reference material is 10 nM. Higher sensitivity may be achieved with other siderophores. RFU: Relative fluorescence units.

**a.**

**
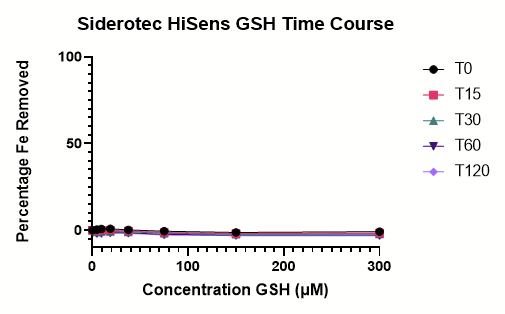
**

**b.**

**
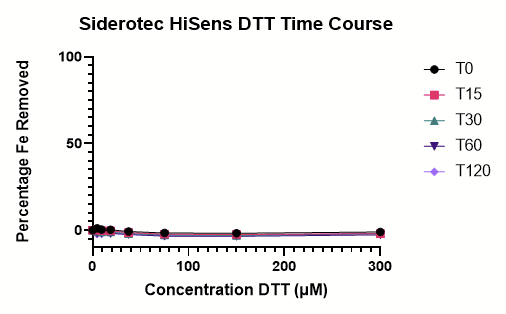
**

**Supplementary Figure 8.** The impact of **a.** glutathione (GSH) and **b.** dithiothreitol (DTT) on the fluorometric  Fe^3+^ assay, Siderotec HiSens over time. The samples were normalized to a percentage relative to the control sample (100%) at each timepoint (min). Neither GSH or TCEP showed any effect on the assay over time (n = 4).

**Adhesion lipoprotein (AdcA)
Uniprot ID: Q82Z67**
**Protein Sequence:**
MKKFTLPLLAALSLILFGACGKTNTSDKTADGKEK**LSIVTTFYPMYDFTK**NIVGDEGDVK**LLIPAGSEPHDYEPSAK**DMATIHDADVFVYHNENMESWVPKAAKGWKKGAPNVIKGTENMVLLPGSDEDGHDHDHEHGEEGHHHELDPHTWVSPHR**AIQEVTNIKEQLVK**LYPKKAKTFETNAEKYLTK**LTALDKEFQTALK**DAKQK**SFVTQHAAFGYLALDYGLKQVPIAGLTPEQEPTAGR**LAELK**KYVTDNQIR**YIYFEKNANDKIAK**TLADEANVQLEVLNPLESLTQKQMDNGEDYLSVMK**ENLTALKKTTDTAGKEVQPETSEKTEK**TVANGYFK**DSEVAER**TLTDYAGNWQSVYPLLKDGTLDQVFDYK**AKLKKDKTPAEYK**TYYDAGYQTDVDHINITDSTIEFLVNGKPQK**FTYKAAGYKILNYAKGNRGVR**FLFETDDANAGR**FK**YVQFSDHNIAPTK**AAHFHIFFGGDSQESLFNEMDNWPTYYPSDLSK**QEIAQEMIAH**

**ABC transporter, ATP-binding protein (AdcC)
Uniprot ID: Q839U4**
**Protein Sequence:**
MHYIEVENLTFYYDDEPVLEDVSYYVDPGEFVILTGENGAAKSTLIK**STLGLLKPTSGK**ITVAKKNSAGEK**ISIGYIPQQVASFNAGFPSTVIELVR**SGRFPRNRWFKPLTKKDHLHVEKALK**SVDMWEMR**HKRIGELSGGQKQRISLAR**VFATDPDLFILDEPTTGMDEQSRNEFYQLLQHSAHEHGKAILMITHDHEDIK**TYVDRQIRLVRKEDSKWR**CFHMSEESYT**

**Adhesion lipoprotein (AdcA-II)
Uniprot ID: Q839U5**
**Protein Sequence:**
MTKIYRRLIIGVTLAISAFLLASCGQTTQSPKEKKELTVMTTFYPMYDFTK**QVVGDEGEVELLIPAGTEPHDYEPSAK**DLAK**ITDADVFVYNSKELETWVPNVIENLDTK**KVSIVEASQSIQLMQGTEEEESGEEGHEGHNHSHELDPHVWLDPVLAQKEVTAIRDALIKKYPEKKAVFEKNTVAYLEK**LTALDKEYQAAFAGAK**NR**TFVTQHAAFGYLAKQYGLTQEPIAGISPDQEPSPSR**LAELKKYIKTNNVSVIYFEASASTKVAK**TLADETGVELAVLNPLESLTQKEQEAGENYVSVMK**ENLAALQKSIH

**Supplementary Figure 9.** The AdcABC zinc uptake system in Gram positive bacteria is functionally equivalent to the ZnuABC system in Gram negative species. AdcA-II is also referred to as adhesion lipoprotein II. Peptides identified by mass spectrometry are highlighted in bold.

**50S ribosomal protein L33 4
Uniprot ID: P59629**
**Protein Sequence:**
**MRQTITLACAETGER**LYLTSKNKRNTPEKLQLKKYSPKLRRR**ALFTEVK**

**50S ribosomal protein L33 3
Uniprot ID: P59628**
**Protein Sequence:**
MR**VNITLE*CTSC*KER**NYLTNKNKRNNPDRLEKQKYCPRERKVTLHRETK

**50S ribosomal protein L28
Uniprot ID: Q82ZE4**
**Protein Sequence:**
MAK**VCYFTGR**KTSSGNNRSHAMNSTKR**TVKPNLQK**VR**VLIDGKPKKVWVSTR**ALKSGKIERV

**50S ribosomal protein L32-3
Uniprot ID: Q836R0**
**Protein Sequence:**
MAVPARRTSKAKKAKRRTHYKLTIKGLNA*CSNC*GEMK**KSHHV*CPAC*GHYDGKDVMSKEA**

**30S ribosomal protein S15
Uniprot ID: Q82ZJ1**
**Protein Sequence:**
MAISQER**KNEIIKEYARHEGDTGSPEVQIAVLTEDINQLNEHAR**THKKDHHSYRGLMKKIGHR**RNLLAYLR**KTDIQR**YRELIQR**LGLRR

**30S ribosomal protein S12
Uniprot ID: Q839H1**
**Protein Sequence:**
M**PTINQLVR**KPRKSKVEK**SDSPALNKGYNSFK**KTQTNVNSPQKRGVCTRVGTMTPKKPNSALRKYARVR**LSNLIEVTAYIPGIGHNLQEHSVVLLR**GGR**VKDLPGVR**YHIVR**GALDTAGVNDR**KQSRSKYGTKRPKA

**50S ribosomal protein L36
Uniprot ID: Q839E1**
**Protein Sequence:**
MK**VRPSVKPM*CEHC*K**VIRRKGR**VMVICPANPK**HKQRQG

**Supplementary Figure 10.** Ribosomal protein amino acid sequences with peptides identified by mass spectrometry highlighted in bold. Highlighted in italics are the possible Zn binding domains (*CxxC*).

**Supplementary Table 1.** All ribosomal proteins altered in abundance when *E. faecalis* was exposed to gliotoxin. Each was searched for a zinc binding domain (CxxC). **Of the 12 ribosomal proteins altered in abundance, 7 do not contain a zinc binding domain, indicating that zinc free paralogs are present upon GT-mediated zinc depletion.** Notably 7/12 ribosomal proteins exhibited altered abundance in a direction commensurate with the presence or absence of a zinc-binding domain and GT presence or absence, and only 5/12 fell into the “contrary” group. Proteins in italics are “contrary” i.e. zinc-binding domain containing ribosomal proteins which are increased in abundance in the presence of GT or vice versa.

**Ribosomal proteins uniquely present with GT at T30**

| **Protein description** | **Peptides** | **Sequence coverage [%]** | **P-value** | **Fold Change (log2)** | **Protein IDs** | **Zn binding domain? (CxxC)** |
| --- | --- | --- | --- | --- | --- | --- |
| 50S ribosomal protein L33 4 | 2 | 40.8 | 1 | NaN | P59629 | No |
|  |  |  |  |  |  |  |

**Ribosomal proteins increased in abundance with GT at T30**

| **Protein description** | **Peptides** | **Sequence coverage [%]** | **P-value** | **Fold Change (log2)** | **Protein IDs** | **Zn binding domain? (CxxC)** |
| --- | --- | --- | --- | --- | --- | --- |
| 30S ribosomal protein S15 | 3 | 39.3 | 0.006185 | 0.690959 | Q82ZJ1 | No |

**Ribosomal proteins decreased in abundance with GT at T30**

| **Protein description** | **Peptides** | **Sequence coverage [%]** | **P-value** | **Fold Change (log2)** | **Protein IDs** | **Zn binding domain? (CxxC)** |
| --- | --- | --- | --- | --- | --- | --- |
| *30S ribosomal protein S12* | *2* | *25.5* | *0.012837* | *-2.08302* | *Q839H1* | *No* |

**Ribosomal proteins uniquely present with GT at T60**

| **Protein description** | **Peptides** | **Sequence coverage [%]** | **P-value** | **Fold Change (log2)** | **Protein IDs** | **Zn binding domain? (CxxC)** |
| --- | --- | --- | --- | --- | --- | --- |
| *50S ribosomal protein L33 3* | *2* | *26.5* | *N/A* | *Unique* | *P59628* | *Yes* |
| 50S ribosomal protein L33 4 | 3 | 44.9 | N/A | Unique | P59629 | No |
| 50S ribosomal protein L28 | 4 | 48.4 | N/A | Unique | Q82ZE4 | No |
| *50S ribosomal protein L32-3* | *4* | *37.3* | *N/A* | *Unique* | *Q836R0* | *Yes* |
|  |  |  |  |  |  |  |

**Ribosomal proteins increased in abundance with GT at T60**

| **Protein description** | **Peptides** | **Sequence coverage [%]** | **P-value** | **Fold Change (log2)** | **Protein IDs** | **Zn binding domain? (CxxC)** |
| --- | --- | --- | --- | --- | --- | --- |
| *50S ribosomal protein L36* | *2* | *60.5* | *1* | *4.49032* | *Q839E1* | *Yes* |

**Ribosomal proteins uniquely present with GT at T180**

| **Protein description** | **Peptides** | **Sequence coverage [%]** | **P-value** | **Fold Change (log2)** | **Protein IDs** | **Zn binding domain? (CxxC)** |
| --- | --- | --- | --- | --- | --- | --- |
| 50S ribosomal protein L33 4 | 2 | 40.8 | 1 | Unique | P59629 | No |
| 30S ribosomal protein S14 2 | 3 | 39.3 | 1 | Unique | Q82Z70 | No |

**Ribosomal proteins uniquely absent with GT at T180**

| **Protein description** | **Peptides** | **Sequence coverage [%]** | **P-value** | **Fold Change (log2)** | **Protein IDs** | **Zn binding domain? (CxxC)** |
| --- | --- | --- | --- | --- | --- | --- |
| 50S ribosomal protein L33 3 | 2 | 26.5 | 1 | Absent | P59628 | Yes |

**Ribosomal proteins which are decreased in abundance with GT at T180**

| **Protein description** | **Peptides** | **Sequence coverage [%]** | **P-value** | **Fold Change (log2)** | **Protein IDs** | **Zn binding domain? (CxxC)** |
| --- | --- | --- | --- | --- | --- | --- |
| *30S ribosomal protein S15* | *6* | *56.2* | *0.008805* | *-1.64109* | *Q82ZJ1* | *No* |

**Supplementary Table 2.A.** Proteins which are **uniquely present** in *E. faecalis* when grown with gliotoxin (5 µg/ml) and Zn (200 µM), compared to identical cultures grown with only gliotoxin (5 µg/ml). Grown using tryptic soy broth (TSB) media for **30 min** in the log phase (0.3-0.4 OD600).

| **Protein description** | **Fold Change (log2)** | **P-value** | **Peptides** | **Sequence coverage [%]** | **Protein IDs** |
| --- | --- | --- | --- | --- | --- |
| PucR family transcriptional regulator | Unique | N/A | 3 | 4.6 | Q82Z01 |
| DNA polymerase III delta N-terminal domain-containing protein | Unique | N/A | 3 | 11.4 | Q831Q4 |
| Ribosomal RNA small subunit methyltransferase E (EC 2.1.1.193) | Unique | N/A | 3 | 13.2 | Q833H7 |
| Phosphate transport system permease protein PstA | Unique | N/A | 2 | 10.5 | Q834B2 |
| Transcriptional regulator, ArsR family | Unique | N/A | 2 | 25.2 | Q839Q2 |
| Serine/threonine transporter SstT (Na(+)/serine-threonine symporter) | Unique | N/A | 3 | 8.6 | Q82ZN5 |
| TPM domain-containing protein | Unique | N/A | 3 | 13 | Q831M4 |
| Undecaprenyl-diphosphatase (EC 3.6.1.27) (Bacitracin resistance protein) (Undecaprenyl pyrophosphate phosphatase) | Unique | N/A | 1 | 5.4 | Q831R1 |
| YxeA family protein | Unique | N/A | 1 | 10.3 | Q832L5 |
| CBS domain protein | Unique | N/A | 3 | 17.5 | Q834D7 |
| Segregation and condensation protein B | Unique | N/A | 3 | 35.3 | Q834U3 |
| histidine kinase (EC 2.7.13.3) | Unique | N/A | 5 | 14.1 | Q835W1 |
| Peptide ABC transporter substrate-binding protein | Unique | N/A | 2 | 9.7 | Q835W7 |
| Pyrroline-5-carboxylate reductase (P5C reductase) (P5CR) (EC 1.5.1.2) (PCA reductase) | Unique | N/A | 2 | 11.8 | Q836Y3 |
| Glycine betaine/carnitine/choline ABC transporter, glycine betaine/carnitine/choline-binding protein | Unique | N/A | 4 | 9.5 | Q837Z8 |
| Oxidoreductase, DadA family | Unique | N/A | 5 | 22.9 | Q838M8 |
| Mini-ribonuclease 3 (Mini-3) (Mini-RNase 3) (EC 3.1.26.-) (Mini-RNase III) (Mini-III) | Unique | N/A | 3 | 33.3 | Q839V4 |
| Cyclic-di-AMP phosphodiesterase (EC 3.1.4.-) | Unique | N/A | 6 | 11.6 | Q839Y7 |

**Supplementary Table 2.B.** Proteins which are **increased in abundance** in *E. faecalis* when grown with gliotoxin (5 µg/ml) and Zn (200 µM), compared to identical cultures grown with only gliotoxin (5 µg/ml). Grown using tryptic soy broth (TSB) media for **30 min** in the log phase (0.3-0.4 OD600).

| **Protein description** | **Fold Change (log2)** | **P-value** | **Peptides** | **Sequence coverage [%]** | **Protein IDs** |
| --- | --- | --- | --- | --- | --- |
| Cadmium-translocating P-type ATPase | 4.09705 | 0.00879968 | 17 | 36.9 | Q835H7 |
| Iron compound ABC transporter, substrate-binding protein | 2.38194 | 0.00196889 | 12 | 47.9 | Q82ZH5 |
| Iron compound ABC transporter, substrate-binding protein | 0.899654 | 0.00552549 | 8 | 36.9 | Q839H9 |
| 30S ribosomal protein S18 | 0.59904 | 0.0356557 | 6 | 44.3 | Q839Y8 |

**Supplementary Table 3.A.** Proteins which are **uniquely absent** in *E. faecalis* when grown with gliotoxin (5 µg/ml) and Zn (200 µM), compared to identical cultures grown with only gliotoxin (5 µg/ml). Grown using tryptic soy broth (TSB) media for **30 min** in the log phase (0.3-0.4 OD600).

| **Protein description** | **Fold Change (log2)** | **P-value** | **Peptides** | **Sequence coverage [%]** | **Protein IDs** |
| --- | --- | --- | --- | --- | --- |
| Transcriptional regulator MraZ | Absent | N/A | 3 | 21.7 | O07103 |
| PTS system, IIA component | Absent | N/A | 3 | 36 | Q831R2 |
| RNA methyltransferase, TrmH family | Absent | N/A | 5 | 31.4 | Q831U7 |
| DNA repair exonuclease family protein | Absent | N/A | 3 | 7.8 | Q837Z2 |
| Holo-[acyl-carrier-protein] synthase | Absent | N/A | 2 | 20.5 | Q820V0 |
| L-serine dehydratase | Absent | N/A | 5 | 33.4 | Q830Q2 |
| Cation efflux family protein | Absent | N/A | 2 | 5.9 | Q837I0 |
| PTS system, IIB component | Absent | N/A | 2 | 15.3 | Q839Y0 |
| DegV family protein | Absent | N/A | 3 | 17 | Q839Y3 |

**Supplementary Table 3.B.** Proteins which are **decreased in abundance** in *E. faecalis* when grown with gliotoxin (5 µg/ml) and Zn (200 µM), compared to identical cultures grown with only gliotoxin (5 µg/ml). Grown using tryptic soy broth (TSB) media for **30 min** in the log phase (0.3-0.4 OD600).

| **Protein description** | **Fold Change (log2)** | **P-value** | **Peptides** | **Sequence coverage [%]** | **Protein IDs** |
| --- | --- | --- | --- | --- | --- |
| Divergent 4Fe-4S mono-cluster domain-containing protein | -1.36149 | 0.0108749 | 6 | 55.3 | Q830Z2 |
| Cadmium-translocating P-type ATPase | -1.15952 | 0.00108666 | 9 | 27.8 | Q830Z1 |
| Asp23/Gls24 family envelope stress response protein | -0.867968 | 0.00703083 | 4 | 70 | Q82ZE5 |
| Alkyl hydroperoxide reductase C | -0.841461 | 0.000564697 | 9 | 52.4 | H7C7A0 |
| Thioredoxin reductase/glutathione-related protein | -0.786518 | 0.000852042 | 23 | 57.3 | Q830N9 |
| Transcriptional regulator, Fur family | -0.76019 | 0.016361 | 6 | 45 | Q834V6 |

**Supplementary Table 4.A**. Comparative detection of proteins which were significantly **uniquely present** with gliotoxin (5 µg/ml) when compared to MeOH controls (left) in the gliotoxin (5 µg/ml) and Zn (200 µM) when compared to gliotoxin (5 µg/ml) proteomics data set (right).

|  |  |  |  |  |  | Gliotoxin and Zn Data | | | |
| --- | --- | --- | --- | --- | --- | --- | --- | --- | --- |
| **Protein description** | **Peptides** | **Sequence coverage [%]** | **P-value** | **Fold Change (log2)** | **Protein IDs** | **Peptides** | **Sequence coverage [%]** | **P-value** | **Fold Change (log2)** |
| Uncharacterized protein | 12 | 36.1 | 1 | NaN | H7C719 | 10 | 28.4 | 0.477189 | 0.938235 |
| 50S ribosomal protein L33 4 | 2 | 40.8 | 1 | NaN | P59629 | 2 | 40.8 | 0.548124 | 0.629477 |
| Cobalamin synthesis protein/P47K family protein | 6 | 25.6 | 1 | NaN | Q82Z69 | 6 | 25.6 | 0.419013 | 1.20201 |
| 30S ribosomal protein S14 1 | 1 | 13.5 | 1 | NaN | Q8KU58 | 1 | 13.5 | 0.47138 | 0.761449 |
| Protein EbsA | 3 | 19 | 1 | NaN | P36920 |  |  |  |  |
| 3-dehydroquinate dehydratase | 3 | 17.4 | 1 | NaN | P36923 |  |  |  |  |
| Uncharacterized protein | 2 | 7.3 | 1 | NaN | Q82YY0 |  |  |  |  |
| Uncharacterized protein | 2 | 18.3 | 1 | NaN | Q82ZK3 |  |  |  |  |
| Transcriptional antiterminator, bglG family | 3 | 8.8 | 1 | NaN | Q82ZT1 |  |  |  |  |
| Xanthine/uracil permease family protein | 2 | 8 | 1 | NaN | Q82ZW2 |  |  |  |  |
| tRNA(Met) cytidine acetate ligase | 3 | 13.3 | 1 | NaN | Q830C2 |  |  |  |  |
| PTS system, beta-glucoside-specific IIABC component | 3 | 6.9 | 1 | NaN | Q831B4 |  |  |  |  |
| Uncharacterized protein | 2 | 55.9 | 1 | NaN | Q834Y7 |  |  |  |  |
| Histidine kinase | 3 | 9.7 | 1 | NaN | Q837B6 | 3 | 9.4 | 0.716151 | -0.0610437 |
| Abhydrolase_3 domain-containing protein | 3 | 14.3 | 1 | NaN | Q838Q5 |  |  |  |  |
| Glyoxylase family protein | 3 | 11.2 | 1 | 0.600289 | Q834I3 |  |  |  |  |

**Supplementary Table 4.B**. Comparative detection of proteins which were significantly **increased in abundance** with gliotoxin (5 µg/ml) when compared to MeOH controls (left) in the gliotoxin (5 µg/ml) and Zn (200 µM) when compared to gliotoxin (5 µg/ml) proteomics data set (right).

|  |  |  |  |  |  | Gliotoxin and Zn Data | | | |
| --- | --- | --- | --- | --- | --- | --- | --- | --- | --- |
| **Protein description** | **Peptides** | **Sequence coverage [%]** | **P-value** | **Fold Change (log2)** | **Protein IDs** | **Peptides** | **Sequence coverage [%]** | **P-value** | **Fold Change (log2)** |
| Adhesion lipoprotein (AdcA-II) | 15 | 39.3 | 0.000462756 | 7.28739 | Q82Z67 | 16 | 48.5 | 0.499674 | 0.716129 |
| ABC transporter, ATP-binding protein (AdcC) | 6 | 35.7 | 0.00277729 | 4.10372 | Q839U4 | 7 | 47 | 0.415357 | 0.781216 |
| Adhesion lipoprotein (AdcABC) | 9 | 45.1 | 0.00528037 | 2.41358 | Q839U5 | 9 | 49.8 | 0.479651 | 0.673272 |
| Transcriptional regulator, Fur family | 4 | 27.8 | 0.00314565 | 1.6883 | Q831T2 |  |  |  |  |
| Acyl carrier protein | 2 | 44.3 | 0.00210808 | 1.27213 | Q82ZE9 | 2 | 44.3 | 0.899965 | 0.0398273 |
| Copper-exporting P-type ATPase | 17 | 29.1 | 0.0166494 | 0.938649 | Q838Y5 | 11 | 20.5 | 0.732133 | 0.114099 |
| Spermidine/putrescine ABC transporter, ATP-binding protein | 2 | 6.9 | 0.0242974 | 0.935763 | Q835Z8 |  |  |  |  |
| Cold shock protein CspC | 4 | 80.3 | 0.0414736 | 0.826335 | Q833G3 | 3 | 78.8 | 0.777506 | -0.137138 |
| Uncharacterized protein | 12 | 23 | 0.0170368 | 0.809572 | Q837F4 | 10 | 18.8 | 0.75499 | 0.0847753 |
| Uncharacterized protein | 3 | 63.8 | 0.0432523 | 0.75339 | Q835R0 | 2 | 50 | 0.052948 | -0.721778 |
| Segregation and condensation protein A | 2 | 9.1 | 0.0318054 | 0.692021 | Q834U4 | 2 | 11.4 | 1 | 0.504814 |
| 30S ribosomal protein S15 | 3 | 39.3 | 0.00618528 | 0.690959 | Q82ZJ1 | 3 | 39.3 | 0.163829 | 0.793812 |
| Thioredoxin | 5 | 79.8 | 0.0330177 | 0.690918 | Q835H2 | 5 | 79.8 | 0.40928 | -0.259165 |
| Uncharacterized protein | 3 | 47.7 | 0.0210995 | 0.688846 | Q830I5 |  |  |  |  |
| Guanosine monophosphate reductase | 8 | 35.1 | 0.030067 | 0.656947 | Q831S1 | 5 | 21.5 | 0.774018 | -0.0554574 |

**Supplementary Table 5.A**. Comparative detection of proteins which were significantly **uniquely absent** with gliotoxin (5 µg/ml) when compared to MeOH controls (left) in the gliotoxin (5 µg/ml) and Zn (200 µM) when compared to gliotoxin (5 µg/ml) proteomics data set (right).

|  |  |  |  |  |  | Gliotoxin and Zn Data | | | |
| --- | --- | --- | --- | --- | --- | --- | --- | --- | --- |
| **Protein description** | **Peptides** | **Sequence coverage [%]** | **P-value** | **Fold Change (log2)** | **Protein IDs** | **Peptides** | **Sequence coverage [%]** | **P-value** | **Fold Change (log2)** |
| Endonuclea_NS_2 domain-containing protein | 2 | 17.4 | 1 | NaN | Q835Q6 | 3 | 23.7 | 0.435554 | -0.227208 |
| ABC transporter, ATP-binding/permease protein | 5 | 12.3 | 1 | NaN | Q837A1 | 7 | 19.1 | 0.56697 | -0.126945 |
| Uncharacterized protein | 3 | 41.7 | 1 | NaN | Q82YV6 |  |  |  |  |
| Lipase, putative | 4 | 15.1 | 1 | NaN | Q82Z80 |  |  |  |  |
| ATP-dependent DNA helicase RecG | 4 | 8.6 | 1 | NaN | Q82ZE7 |  |  |  |  |
| YitT family protein | 3 | 12.4 | 1 | NaN | Q82ZG6 | 3 | 17.1 | 1 | -0.38885 |
| 3-hydroxyacyl-[acyl-carrier-protein] dehydratase FabZ ((3R)-hydroxymyristoyl-[acyl-carrier-protein] dehydratase) (Beta-hydroxyacyl-ACP dehydratase) | 2 | 27.3 | 1 | NaN | Q833N7 |  |  |  |  |
| Phosphate transport system permease protein PstA | 2 | 7.1 | 1 | NaN | Q834B2 | 2 | 10.5 | 1 | NaN |
| ABC transporter, ATP-binding protein | 3 | 23.2 | 1 | NaN | Q835Q5 |  |  |  |  |
| PTS system, IIA component | 3 | 50 | 1 | NaN | Q836T9 |  |  |  |  |
| DUF2200 domain-containing protein |  | 49.1 | 1 | -0.62912 | Q833J9 |  |  |  |  |
| YlbF family regulator | 2 | 20.1 | 1 | -1.00102 | Q831P6 |  |  |  |  |

**Supplementary Table 5.B**. Comparative detection of proteins which were significantly **decreased in abundance** with gliotoxin (5 µg/ml) when compared to MeOH controls (left) in the gliotoxin (5 µg/ml) and Zn (200 µM) when compared to gliotoxin (5 µg/ml) proteomics data set (right).

|  |  |  |  |  |  | Gliotoxin and Zn Data | | | |
| --- | --- | --- | --- | --- | --- | --- | --- | --- | --- |
| **Protein description** | **Peptides** | **Sequence coverage [%]** | **P-value** | **Fold Change (log2)** | **Protein IDs** | **Peptides** | **Sequence coverage [%]** | **P-value** | **Fold Change (log2)** |
| 30S ribosomal protein S12 | 2 | 25.5 | 0.0128374 | -2.08302 | Q839H1 | 3 | 31.4 | 0.240086 | -0.602329 |
| Sodium/dicarboxylate symporter family protein | 11 | 23.5 | 0.0460545 | -0.929064 | Q837T6 | 11 | 26.6 | 0.391498 | 0.169872 |
| Cysteine synthase B, putative | 10 | 49.8 | 0.0200332 | -0.843203 | Q838Z3 | 9 | 49.8 | 0.443246 | -0.117821 |
| Cyclopropane-fatty-acyl-phospholipid synthase | 15 | 54.9 | 0.0158166 | -0.711893 | Q839G6 | 14 | 43.3 | 0.730173 | -0.132547 |
| Magnesium-transporting ATPase, P-type 1 | 16 | 23.7 | 0.0195624 | -0.63794 | Q835M5 | 13 | 20.3 | 0.559754 | 0.0611273 |

**Supplementary Table 6**. Decreased abundance of AdcABC system proteins in *E. faecalis* treated with gliotoxin (5 µg/ml) and Zn (400 µM) compared to gliotoxin (5 µg/ml) only after 60 min.

| **Protein description** | **Peptides** | **Sequence coverage [%]** | **P-value** | **Fold Change (log2)** | **Protein IDs** |
| --- | --- | --- | --- | --- | --- |
| Adhesion lipoprotein (AdcA-II) | 17 | 44.4 | 0.182046 | -1.4965 | Q82Z67 |
| ABC transporter, ATP-binding protein (AdcC) | 6 | 35.7 | 0.178631 | -1.31166 | Q839U4 |
| Adhesion lipoprotein (AdcABC) | 10 | 50.8 | 0.117702 | -1.45304 | Q839U5 |

**Supplemental Table 7.** Determination of the pKd of Zn^2+^ complex of the metal chelators DTG (8.72) and TPEN (9.69) using PAR at pH 7.4.

| **Sample** | **L_total_ (µM)** | **A_492_** | **[ZnHx(PAR)_2_] (µM)** | **[PAR] (µM)** | **[L] (µM)** | **[ZnL] (M)** | **K_ex_ (M)** | **K_d_^ZnL^ (M)** | **pKd** | **Average pKd of 10 and 5 uM** |
| --- | --- | --- | --- | --- | --- | --- | --- | --- | --- | --- |
| Blank | 0 | 0.539833 | 7.5501 | 84.89977 |  |  |  |  |  |  |
| DTG 10 µM | 10 | 0.315333 | 4.4103 | 91.17949 | 6.8601 | 3.14E-06 | 8.63E-04 | 8.21E-10 | 9.09 |  |
| DTG 5 µM | 5 | 0.4935 | 6.9021 | 86.1958 | 4.3520 | 6.48E-07 | 1.60E-04 | 4.42E-09 | 8.35 | 8.72 |
| TPEN 10 µM | 10 | 0.2595 | 3.6294 | 92.74126 | 6.0793 | 3.92E-06 | 1.53E-03 | 4.63E-10 | 9.33 |  |
| TPEN 5 µM | 5 | 0.263167 | 3.6807 | 92.63869 | 1.1305 | 3.87E-06 | 7.98E-03 | 8.87E-11 | 10.05 | 9.69 |
